# SERS spectral study of HAuCl_4_-cysteine nanocatalytic reaction and its application for detection of heparin sodium with label-free VB4r molecular probe

**DOI:** 10.1038/srep45979

**Published:** 2017-04-05

**Authors:** Xiaoliang Wang, Caina Jiang, Yanna Qin, Yutao Peng, Guiqing Wen, Aihui Liang, Zhiliang Jiang

**Affiliations:** 1 Key Laboratory of Ecology of Rare and Endangered Species and Environmental Protection of Ministry Education, Guangxi Key Laboratory of Environmental Pollution Control Theory and Technology, Guangxi Normal University, Guilin 541004, China

## Abstract

In the presence of nanocatalyst, *L*-cysteine reduce HAuCl_4_ rapidly to form gold nanoparticles (AuNP), and a quick nanocatalytic preparation procedure was established for Au/AuNP sol with highly active surface enhanced Raman scattering (SERS) effect and good stability. The nanoreaction was also studied by absorption, resonance Rayleigh scattering (RRS), transmission electron microscopy (TEM) and energy spectra. In the selected conditions, the analyte heparin sodium (HS) could react with victoria blue 4 R (VB4r) to form associated complexes which have very weak SERS effect to make the SERS signals decrease. The SERS signals at 1617 cm^−1^ reduced linearly with HS concentration increasing. Upon addition of FeCl_3_, it hydrolyzed to form stable Fe(OH)_3_ sol platform that carried SERS active Au/AuNPs to enhance the sensitivity. Accordingly, we established a SERS quantitative analysis method in the sol substrate of Fe(OH)_3_-Au/AuNPs, with a linear range of 0.5–75 ng/mL HS and a detection limit of 0.2 ng/mL. HS in real samples was determined, with a relative standard deviation of 2.65–7.63% and a recovery of 99.3–101%.

Surface-enhanced Raman scattering (SERS) spectroscopy was a kind of sensitive molecular spectral analysis technology, and the sensitivity was closely related to its substrate. Gold and silver nanoparticles have good SERS enhancement effect, simple preparation and easy store, and they were commonly used SERS substrates. At present, chemical and physical methods were used for preparation of gold and silver nanoparticles. The most commonly chemical method is liquid-phase, which prepared nanoparticles using appropriate reducer, with flexibllty and easy controlling of the morphology and size. However, some of the methods need to use harmful chemicals and there may be a potential pollution to the environment. In recent years, environmental friendly green synthesis of nanoparticles has got more and more extensive research^1^,^2^,^3^,^4^,^5^. Yang *et al*.^1^ reported a simple seed-mediated method to synthesize silver nanoparticles in size of 50–300 nm. Kim *et al*.^2^ prepared the gold nanoparticles with caffeic acid as reducer, and the particle geometry could be controlled effectively by changing the caffeic acid concentration. Although silver nanoparticles commonly had higher SERS activity than gold nanoparticles, its stability was less than gold nanoparticles, and it was easier to be oxidized^6^,^7^,^8^,^9^,^10^. Therefore, the preparation of gold nanoparticle with high SERS activity was very important to SERS quantitative analysis. Lopez *et al*.^6^ developed a simple and sensitive SERS detection immunoassay based on antigen-mediated aggregation of gold nanoparticles with a detection limit of 1.9 ng/mL, and it was 20-fold higher than the ELISA assay. Lee *et al*.^7^ reported a solution-based method on chip by growth of wrinkled gold nanoparticles. Using the fabricated substrate, a SERS enhancement was 30-times stronger than the spherical nanoparticle substrate. Li *et al*.^8^ fabricated a SERS sensor for the first time to detect aflatoxin B1 based on gold nanostar core–silver nanoparticle satellites. Zhang *et al*.^9^ established a biosensor for *salmonella typhimurium* and *staphylococcus aureus* based on gold nanoparticle substrate and the specific recognition of aptamer. Ye *et al*.^11^ explained the nanogold catalysis in the synthesis of gold nanosol and the possibility of improving the SERS detection sensitivity. Wen *et al*.^12^ reported a novel SERS method for trace human chorionic gonadotropin, based on the catalytic effect of nanosilver on the H_2_O_2_-HAuCl_4_ nanoreaction. Up to date, there are no reports about the green and rapid preparation method for high SERS active gold nanosol, based on the AuNP catalysis of *L***-**cysteine-HAuCl_4_ nanoreaction, and using the gold nanosol substrate to SERS quantitative analysis of trace analyte.

Heparin Sodium (HS) is a polysaccharide sulfate type of anticoagulants, and is a kind of amino dextran sulfate sodium from extraction in the intestinal mucosa of pigs or cows. Since the effect of anticoagulant^13^,^14^,^15^, falling hematic fat, protecting endothelial cells and platelet accumulation and release^16^,^17^,^18^, promoting fibrinolysis, inhibiting artery smooth muscle cell proliferation, reducing blood viscosity and anti-inflammatory effect, it has been used in clinical drugs, and also has a significant role in the field of molecular biology^19^,^20^,^21^. Although heparin for the prevention and treatment of thromboembolic disease has very effect, it still needs to be used carefully in medicine. The study found that long-term taking heparin drugs may be inhibitory effect on angiogenesis^22^,^23^,^24^. Particularly, the maternal and other people who have various kinds of diseases such as hemorrhagic diathesis and accompanied blood clotting delay are banned to use it. And taking HS excessively could lead to sudden bleeding, thrombocytopenia, and adverse reactions such as allergic inflammation. So it is great significance to explore a new sensitive, selective method for determination of HS. At present, there are two main types of methods for HS. One type was biological method^25^,^26^,^27^, based on its anticoagulant activity. The detection result was good, but the operation was complex, and detecting cost was high. The other type was chemical method, mainly included chromatography^28^,^29^,^30^, fluorescence^31^,^32^,^33^, spectrophotometry^34^,^35^ and so on. Although the operation was simple, the results were greatly influenced by many factors. Up to date, SERS quantitative analysis of heparin sodium has not been reported. In this paper, the *L*-cysteine-HAuCl_4_ nanoreaction was studied to prepare AuNP nanosol with high SERS activity. A simple, rapid, sensitive and selective SERS quantitative analysis method was developed for detection of HS in the as-prepared AuNP sol substrate, using label-free molecular probe of VB4r.

## Results

### Transmission electron microscopy (TEM) and Energy spectra (EDS)

**Figure 1:**
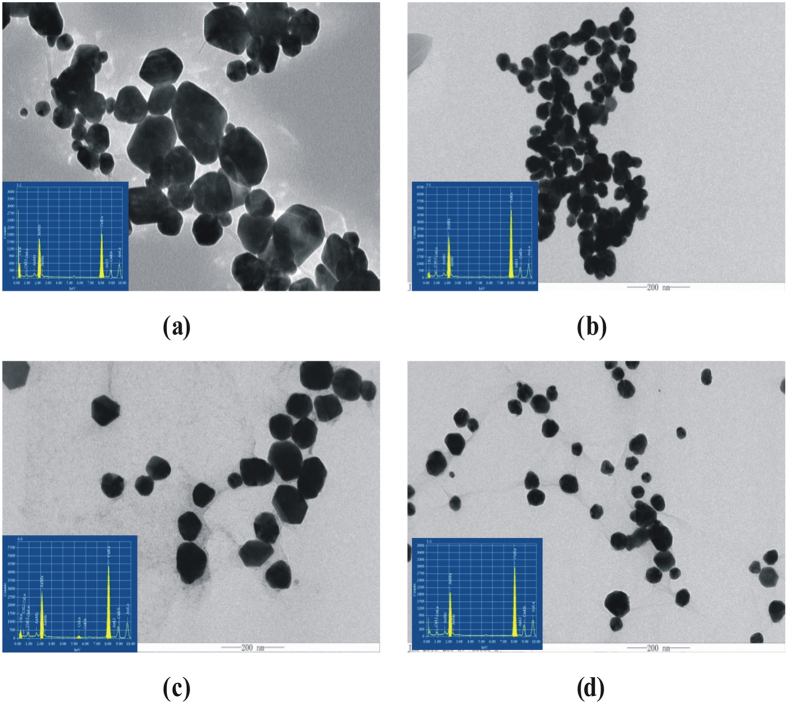
TEM and EDS of AuNPs. (**a**) 0.1 mmoL/L *L*-CyS + 0.5 mL 1% HAuCl_4_, 50 °C for 5 min; (**b**) a + 0.58 μg/mL AuNPc; (**c**) a + 1.9 μmoL/L AgNPs; (**d**) a + 0.5 mL 0.02% GO.

The transmission electron microscopy (TEM) of the 4 kinds of as-prepared nanoparticles (see SI) was recorded. We tried to prepare AuNPs by low concentration of *L-*CyS at room temperature and at 50 °C respectively. The former of AuNPs was irregular shape, and the latter was quasi-sphere particles [Fig. 1]. The small AuNPs were prepared, based on AuNPc, AgNPs and graphene oxide (GO) catalyzing the CyS-AuCl_4_^−^ reaction at 50 °C [Fig. 1]. The results showed that the reaction was speeded up by the three nanocatalysts that enhance the redox electron transfer between CyS and AuCl_4_^−^ to form more nucleuses to grow more small nanoparticles with high SERS activity.

The energy spectra of AuNP system were recorded by using transmission electron microscope with 200kv. The EDS of AuNPs produced by the *L-*cysteine, AuNPc, AgNPs, and GO were obtained respectively [Fig. 1]. As the spectra showed, there are three peaks at 1.7, 2.1 and 9.7 keV for Au element, the composition and concentration ratio of the four kinds of AuNPs were nearly the same. So, it can be concluded that the three nanoparticles of AuNPc, AgNPs and GO had the effect of catalyst, and did not generate composite nanomaterials.

### Ultraviolet absorption, RRS and SERS spectra

The ultraviolet absorption spectra of gold nanosol were recorded. As shown in Fig. S1, AuNPs appeared a wide surface Plasmon resonance (SPR) absorption peak at 560 nm. The SPR peak of Au/AuNPs, Ag/AuNPs and GO/AuNPs appeared at 550 nm, 550 nm and 560 nm respectively [Fig. S1]. The SPR peak changes were due to the difference of morphology and size. And the peaks height increased with the increase of nanoparticle concentration. The absorption spectra of analysis systems were studied [Fig. S2]. With increase of HS concentration, the characteristic peak at about 550 nm increased gradually. The reason was that VB4r could combine with nanoparticles to decrease their absorption value. When HS existed, it reacted with VB4r to release AuNPs that caused the absorption peak increasing. Due to the similar morphology and uniform particle size of the dispersed nanoparticles, the absorption peak became narrower.

**Figure 2:**
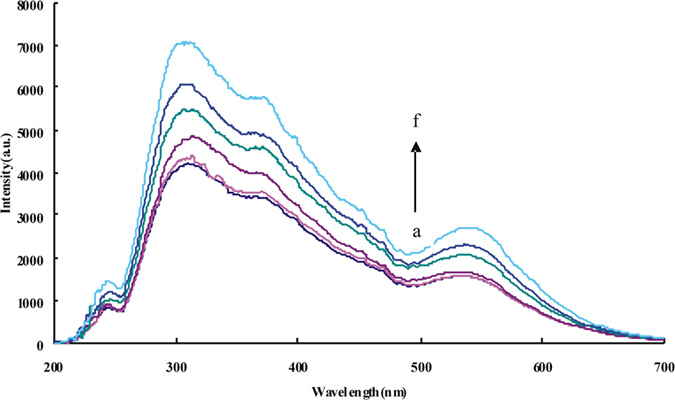
RRS spectra of Au/AuNPs-HS-VB4r system. (**a**) pH 4.0 20 mmoL/L NaAc-HAc + 0.195 mmoL/L Au/AuNPs + 0.1 μmoL/L VB4r; (**b**) a + 0.5 ng/mL HS; (**c**) a + 5 ng/mL HS; (**d**) a + 50 ng/mL HS; (**e**) a + 100 ng/mL HS; (**f**) a + 150 ng/mL HS.

The RRS spectra of AuNPs were investigated. They had two RRS peaks at 370 nm and 550 nm [Fig. S3]. The intensity of the two peaks increased gradually with the increased of AuNP concentration, but when the concentration reached a certain level, the RRS peak around 550 nm began to decline, and had a tendency to widen. This is because the multiple scattering occurred between the nanoparticles, and resuted in the scattering quenching. The RRS spectra of analysis system were studied [Fig. 2 and Fig. S4(a–c)]. The RRS intensity at 310 nm, 370 nm and 550 nm enhanced gradually with increase of HS concentration, because HS react with VB4r to generate VB4r-HS associated molecules and (VB4r-HS)_n_ association particles that exibited strong RRS effect

**Figure 3:**
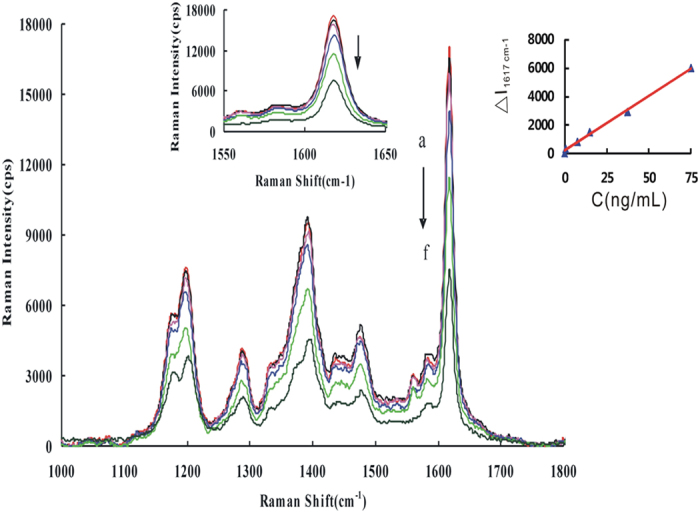
SERS of Au/AuNPs-HS-VB4r-FeCl_3_ System. (**a**) pH 4.0 20 mmoL/L NaAc-HAc + 0.1 μmoL/L VB4r + 0.195 mmoL/L Au/AuNPs + 1.5 μmoL/L FeCl_3_; (**b**) a + 0.5 ng/mL HS; (**c**) a + 5 ng/mL HS; (**d**) a + 50 ng/mL HS; (**e**) a + 100 ng/mL HS; (**f**) a + 150 ng/mL HS.

The SERS effect of AuNPs, Au/AuNPs, Ag/AuNPs and GO/AuNPs with VB4r as molecular probe was studied [Fig. S5]. The SERS peaks appeared at 1184 cm^−1^, 1197 cm^−1^, 1386 cm^−1^, 1477 cm^−1^, 1617 cm^−1^ and so on, and the peak 1617 cm^−1^ was the strongest that ascribed to the bending vibration of C = N and C = C^10^. With the increase of the probe concentration, the SERS signal increased, and a linear relationship was obtained. The SERS effect of VB4r molecular probe was studied in the AuNPs, Au/AuNPs, Ag/AuNPs and GO/AuNPs analytical systems respectively [Fig. 3 and Fig. S6]. The four systems all exhibited SERS peaks at 1182 cm^−1^, 1198 cm^−1^, 1386 cm^−1^, 1477 cm^−1^ and 1617 cm^−1^. The strongest SERS peak located at 1617 cm^−1^ that was ascribed to the bending vibration of C = N and C = C. The Au/AuNPs sol substrate was more sensitive than the others, and was chosen for use. The SERS spectra of Au/AuNPs-VB4r-HS-FeCl_3_, Au/AuNPs-VB4r-HS-AlCl_3_ and Au/AuNPs-VB4r-HS analytical systems were recorded respectively. All systems exhibited peaks at 1182 cm^−1^, 1198 cm^−1^, 1386 cm^−1^, 1477 cm^−1^ and 1617 cm^−1^, and the most sensitive peak at 1617 cm^−1^ all decreased with the HS concentration increasing [Fig. 3 and Fig. S6]. The Au/AuNPs-VB4r-HS-FeCl_3_ analytical system and the peak at 1617 cm^−1^ was the most sensitive and stable, and were selected to use.

### Optimization of AuNP preparing conditions

One purpose of this paper was to prepare a kind of gold nanosol with high SERS activity, so the stable nanosol with high SERS activity was expected. Acorrding to the procedure, the effect of *L-*CyS concentration was studied. The SERS value at *I*_*1617cm−1*_ increased rapidly with the increase of *L-*CyS concentration, and then increased slowly (Fig. S7). Because the *L-*CyS reduced HAuCl_4_ to produce nanoparticles quickly at first and cause SERS effect rapidly increase. With *L-*CyS increase, the reaction trend to balance and the SERS effect increased slowly. The SERS values at *I*_*1617cm-1*_ of AuNPs prepared by different *L-*CyS concentrations were detected for five days (Table S1). The relative standard deviation (RSD) was the lowest when the *L-*CyS concentration was 0.2 mmoL/L. As reducing agent, more *L-*CyS can produce more nanoparticles and make SERS signals increasing, but too high concentration of *L-*CyS produce big and unstable nanoparticles. So a 0.2 mmoL/L *L-*CyS was selected. And the effect of catalyst concentration was studied [Fig. S8]. The corresponding concentrations of AuNPc, AgNPs and GO were 0.58 μg/mL, 4 nmoL/L and 2 μg/mL respectively. The effect of temperature was studied. The SERS signal of *I*_*1617cm-1*_ was maximum at the temperature of 50 °C (Fig. S9). So a temperature of 50 °C was selected. The effect of reaction time was studied. The SERS signal of *I*_*1617cm-1*_ was optimal at 5 min (Fig. S10). So, a reaction time was 5 min. The stability of AuNP sol was examined. The 4 kinds of prepared nanosol were stored at room temperature for several days, and the results of spectral characterization were determined at different time (Tables S2–S5). Their RSDs were less than 10% within 7 days, so the prepared nanoparticles were relatively stable.

The common preparing methods of goldnanoparticle include sodium citrate, sodium borohydride, ascorbic acid, microwave, photochemical and microorganism methods^36^,^37^,^38^,^39^,^40^,^41^,^42^ (Table S6). Sodium citrate method is simple, but the SERS activity is low. The method of this paper is fast, efficient, stable, uniform, and the nanoparticles have high SERS activity.

### Optimization of analysis conditions

**Figure 4:**
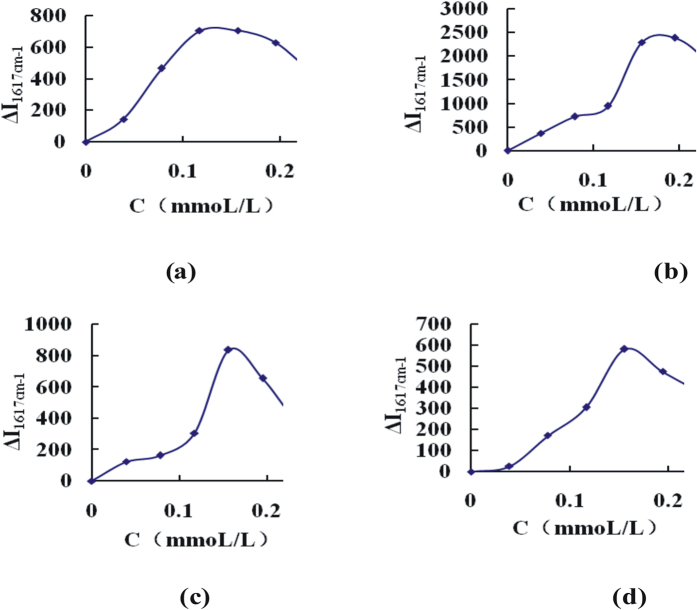
Effect of nanosol substrate concentration on the ΔI_1617cm-1_ 5 mmoL/L pH4.0 NaAc-HAc + nanoparticles + 0.25 μmoL/L VB4r + 100 ng/mL HS. (**a**) AuNPs; (**b**) Au/AuNPs; (**c**) Ag/AuNPs; (**d**) GO/AuNPs.

The effects of substrate AuNPs, Au/AuNPs, Ag/AuNPs and GO/AuNPs concentration on ΔI_1617cm-1_ were investigated [Fig. 4]. According to the results, a 0.156 mmoL/L AuNPs, 0.156 mmoL/L Ag/AuNPs, 0.156 mmoL/L GO/AuNPs and 0.195 mmoL/L Au/AuNPs, formed the most stable and strongest SERS active nanosol and given highest SERS value, were selected to use respectively. The effect of pH value and NaAc-HAc concentration on ΔI_1617cm-1_ was investigated respectively. When the pH achieved at 4.0, ΔI was the largest, and pH 4.0 was chosen (Fig. S11). When the buffer solution concentration was 4 mmol/L, the ΔI value was the largest and a 4 mmol/L NaAc-HAc buffer solution was chosen (Fig. S12). The effect of VB4r concentration on ΔI_1617cm-1_ was examined (Fig. S13). When VB4r concentration was 0.1 μmol/L, the ΔI was the largest, and a 0.1 μmol/L VB4r was chosen. The effect of AlCl_3_ and FeCl_3_ as sensitizer on the system ΔI_1617cm-1_ was considered. When AlCl_3_ and FeCl_3_ concentration was 1.5 μmol/L and 1.5 μmol/L FeCl_3_ respectively, ΔI_1617cm-1_ was the largest (Fig. S14). So 1.5 μmol/L AlCl_3_ and 1.5 μmol/L FeCl_3_ were selected for use.

Six standard curves of different detection systems were obtained according to the procedure [Fig. S15]. We can see that the system of Au/AuNPs-HS-VB4r-Fe^3+^ is the most sensitive (Table 1). The HS concentration had a good linear relationship with SERS intensity Δ*I*_*1613cm-1*_ in the range of 0.5–75 ng/mL, with a detection limit of 0.2 ng/mL, and was selected for use. Comparing the sensitivity of the reported methods for HS (Table 2), the new SERS quantitatiuve analysis method is simple, sensitive and selective.

The influences of common coexistence on the detection of 0.25 μg/mL HS were investigated according to the procedure (Table S7). The results shown that when the relative error were within ± 10%, 100 times Cr^6+^, Pb^2+^, K^+^, Zn^2+^, Mn^2+^, Ca^2+^, Co^2+^, Mg^2+^, Ba^2+^, NO_2_^−^, glycine, *L-*valine, *L-*threonine, lauric acid, etearic acid, leithin, and cetyltrimethyl ammonium bromide, 10 times Ni^2+^, Bi^3+^, and oxalic Acid within, 1 time S_2_O_3_^2−^ and SO_3_^2−^ did not interfered the dection. A 10 times Fe^3+^ did not disturb the detection with 25 μmol/L ascorbic acid masking. Therefore, the method had a good selectivity.

### Sample analysis

The HS contents in HS injection (6250 IU/mL) produced by three pharmaceutical companies of China were analyzed. The known HS was added to the sample and the recovery was tested according to the procedure. Results (Table S8) showed that the RSD was 2.65–7.63% and the recovery was 99.3–101%.

## Discussion

### Analytical and catalytic reaction principles

**Figure 5:**
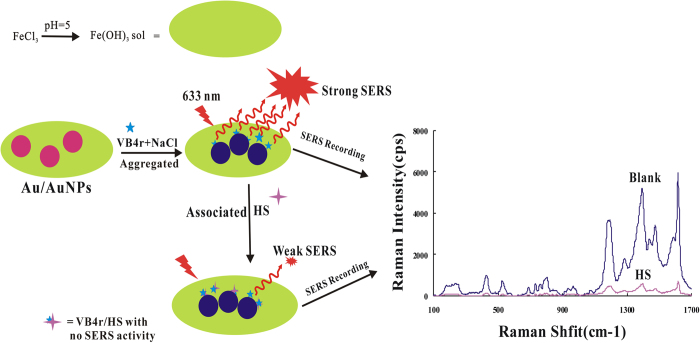
Scheme of SERS detection principle.

In the four SERS nanosol substrates, the Au/AuNPs is most sensitive and was selected for use. Upon addition of FeCl_3_ in the system, a big and stable Fe(OH)_3_ sol formed and combined with Au/AuNPs to produce highly active SERS substrate of Fe(OH)_3_-Au/AuNPs due to more hot-spots on the platform. The molecules of HS contained sulfate radical, which could be hydrolyzed to negatively charged ions in the water, then reacted with positively charged amino in VB4r molecules that adsorbed on the platform, to form VB4r-HS associated molecules and associated particles by means of hydrophobic and intermolecular forces, and led to probe concentration decrease linearly (Fig. 5). In certain conditions, the analyte HS reduced the adsorption of molecular probe on the nanosurface because of the VB4r-HS associated reaction. With the increase of HS concentration, the adsorption quantity of molecular probe decreased, this would result in SERS intensity decreased linearly. Thus, a new SERS quantitative analysis method was set up for detecting HS.

**Figure 6:**
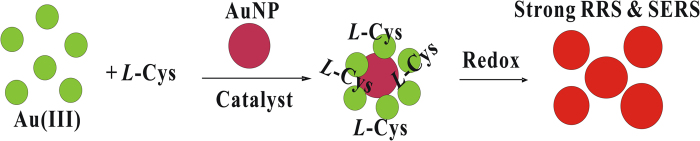
Schematic diagram of generating AuNP_S_.

Due to their small size, large specific surface area and surface free electrons, nanoparticles can be used as intermedium of electron gain-loss of redox reaction, and then accelerated the reaction. Thereby, nanoparticles in redox reaction have very high catalytic activity. In solution system, *L-*CyS can reduce AuCl_4_^−^ directly to generate elemental gold (Au^3+^ + 3e^−^ → Au), but the reaction is slow because it was weak reducer. *L-*CyS reduced Au(III) to form Au(I), and then reduced to Au, and Au(I) was the pivotal intermediate for the synthesis of gold nanoparticles^36^. Upon addition of AuNPs as catalyst, it enhanced the electron transfer, rapidly produced large number of Au(I), and small AuNPs were obtained quickly. The nanosol prepared by nanocatalyst AuNPc was stabilized, and has strong Rayleigh scattering effect. When the probe such as VB4r was added, it showed a great SERS effect (Fig. 6).

## Conclusion

In summary, a highly active SERS effect AuNP sol was prepared by *L*-cysteine reducing HAuCl_4_ and using trace nanoparticles as nanocatalyst at 50 °C water bath. The related nanocatlysis systems and the analysis systems for HS have been investigated by SERS, TEM, EDS, absorption and RRS spectra techniques. According to the results, a new SERS method for HS had been developed by using VB4r as molecular probe in Fe(OH)_3_-Au/AuNP nanosol substrate, with simplicity, high sensitivity and selectivity. The new method was used to analyze HS in real samples with satisfactory results. What’s more, the the analysis mechanism was discussed.

## Methods

### Apparatus and reagents

A model of DXR smart Raman spectrometer (Thermo Company, United States) with laser wavelength of 633 nm and power of 3.0 mW, Cary Eclipse fluorescence spectrophotometer (Varian Company, United States), a model of TU-1901 double beam uv-visible spectrophotometer (Beijing General Instrument Co., LTD, China), and C-MAG HS7 heating magnetic stirrer (IKA Company, Germany) were used. A model of JEM-2100 field emission transmission electron microscope (The Japanese electronics) was used to record the TEM and energy spectrum, with dot resolution of 0.19 nm, line resolution of 0.14 nm, accelerated voltage of 200 kV and tilt angle of 25 degrees.

A 0.01 moL/L *L-*cysteine (*L-*2-amino-3-mercaptopropionic acid, *L-*CyS), 1% chloroauric acid, 10 mg/mL heparin sodium, and 0.1 mmoL/L Victoria blue 4 R (VB4r) were prepared. All reagents are analytically pure, and water was double-distilled.

### Procedure

A 200 μL pH 4.0 NaAc-HAc buffer solution, 100 μL 2.0 μmoL/L VB4r, and a certain amounts of 0.1 mg/mL HS were mixed together. After reacted for about 5 minutes, 1.0 mL 0.39 mmoL/L nanosol and 100 μL 30 μmoL/L FeCl_3_ were added before diluting to 2.0 mL and mixed well. Then, the mixture was transferred into a quartz cell, and recorded the SERS spectra. The SERS peak intensity *I* at 1617 cm^−1^ and the *I*_0_ blank without HS were recorded, and the Δ*I* = *I*–*I*_0_ was calculated.

## Additional Information

**How to cite this article:** Wang, X. *et al*. SERS spectral study of HAuCl_4_-cysteine nanocatalytic reaction and its application for detection of heparin sodium with label-free VB4r molecular probe. *Sci. Rep.* **7**, 45979; doi: 10.1038/srep45979 (2017).

**Publisher's note:** Springer Nature remains neutral with regard to jurisdictional claims in published maps and institutional affiliations.

## Supplementary Material

Supplementary Information

## Figures and Tables

**Table 1:** Comparison of analysis features of SERS methods for HS.

System	Regression Equation	Linear Range (ng/mL)	Coefficient R^2^	Detection limit (ng/mL)
AuNPs	Δ*I*_*1617cm-1*_ = 24.9 C + 35.8	5–200	0.992	3
Au/AuNPs	Δ*I*_*1617cm-1*_ = 40.8 C + 153	1.25–150	0.9976	0.6
Au/AuNPs-Al^3+^	Δ*I*_*1617cm-1*_ = 44.2 C + 277	1.25–150	0.9902	0.5
Au/AuNPs-Fe^3+^	Δ*I*_*1617cm-1*_ = 76.9 C + 182	0.5–75	0.9969	0.2
Ag/AuNPs	Δ*I*_*1614cm-1*_ = 19.6 C + 143	5–200	0.991	3
GO/AuNPs	Δ*I*_*1613cm-1*_ = 20.1 C + 14.9	2.5–150	0.9952	1

**Table 2:** Comparison of reported methods for HS.

Method	Theory	Linear Range	Detection limit	Annotation	Refs
RFS	HS reacted with sulfur to form ionic association, which generated strong fluorescence resonance Rayleigh scattering.	0.025–0.5 μg/mL	—	simple operation but less sensitivity	33
RRS	Heparin integrated with concanavalin A, then hydrogen bond and the solid liquid interface induced RRS enhancement with electrostatic attraction.	8.28–2500 ng/mL	2.48 ng/mL	Sensitive, high accuracy, poor stability, difficult operation	35
AELC	An anion-exchange liquid chromatography method for the determination of heparin and its impurities was developed using chemometric assisted optimization.	—	7.2 μg/mL	Simple operation but less accuracy	28
SERS	SERS signals was reduced by HS reaction with VB4r	0.5–100 ng/mL	0.2 ng/mL	Simple, rapid, sensitive, selective	The paper
