# Supplementary material for: SERS spectral study of HAuCl4-cysteine nanocatalytic reaction and its application for detection of heparin sodium with label-free VB4r molecular probe
Source: Sci Rep. 2017 Apr 5;7:45979. doi: 10.1038/srep45979 (PMC5380991; doi:10.1038/srep45979)
Supplement: Supplementary Information [file srep45979-s1.pdf]

# SERS spectral study of $HAuCl_4$-cysteine nanocatalytic reaction and its application for detection of heparin sodium with label-free VB4r molecular probe

Xiaoliang Wang[§], Caina Jiang[§], Yanna Qin[§], Yutao Peng, Guiqing Wen, Aihui Liang[*], Zhiliang Jiang[*]

Key Laboratory of Ecology of Rare and Endangered Species and Environmental Protection of Ministry Education, Guangxi Key Laboratory of Environmental Pollution Control Theory and Technology, Guangxi Normal University, Guilin 541004, China

**Preparation of nanoparticles.**

**AuNPc**: it was synthesized through reduction of $HAuCl_4$ by trisodium citrate. 50 mL water was added into a flask, heated to boil. Then 0.5 mL 1% $HAuCl_4$ and 3.5 mL 1% trisodium citrate were added rapidly into the boiling water successively. After boiling for 10 min with stirring, the color became from colorless to wine red. The mixture was continued stirring to room temperature, and then diluted to 50 mL. The AuNPc concentration was 58 μg/mL, with a size of about 10 nm.

**AgNPs**: A 0.19 mmol/L yellow silver nanotriangle (AgNPs) was prepared as follows, a 40 mL water, 3.5 mL 1% trisodium citrate and 385 μL $2.4\times10^{-2}$ moL/L $AgNO_3$ were added into a triangle flask in turn with stirring. Then a 4.0 mL 0.5 mg/mL $NaBH_4$ was dripped slowly. After 20 min, the yellow AgNPs can be obtained. After diluting to 50 mL, it was stored at 4℃.

**GO**: Graphene oxide (GO) was prepared by Hummer procedure, and 0.1 mg GO was dissolved in 100 mL double-distilled water with ultrasonicing, the GO solution was 10 μg/mL. It was ultrasonicing 15 min before use.

**Preparation of AuNPs with nanocatalysis**: A 49 mL water, 500 μL 0.01 moL/L *L*-CyS, and 500 μL 1% $HAuCl_4$ were added into a triangle flask, shook well, then reacted in 50℃ water for about 5 min. The solution color changed from canary to colourless, then to bronze, and the AuNPs was obtained, with the concentration of 0.39 mmoL/L. Repeated the above steps, and added 0.5 mL 58 μg/mL AuNPc or 0.5 mL 0.4 mmoL/L AgNPs or 1 mL 5μg/mL GO into the reaction solution. Then nanoparticles Au/AuNPs, Ag/AuNPs or GO/AuNPs could be obtained by the nanocatalytic procedure, and all their concentration were 0.39 mmoL/L Au.

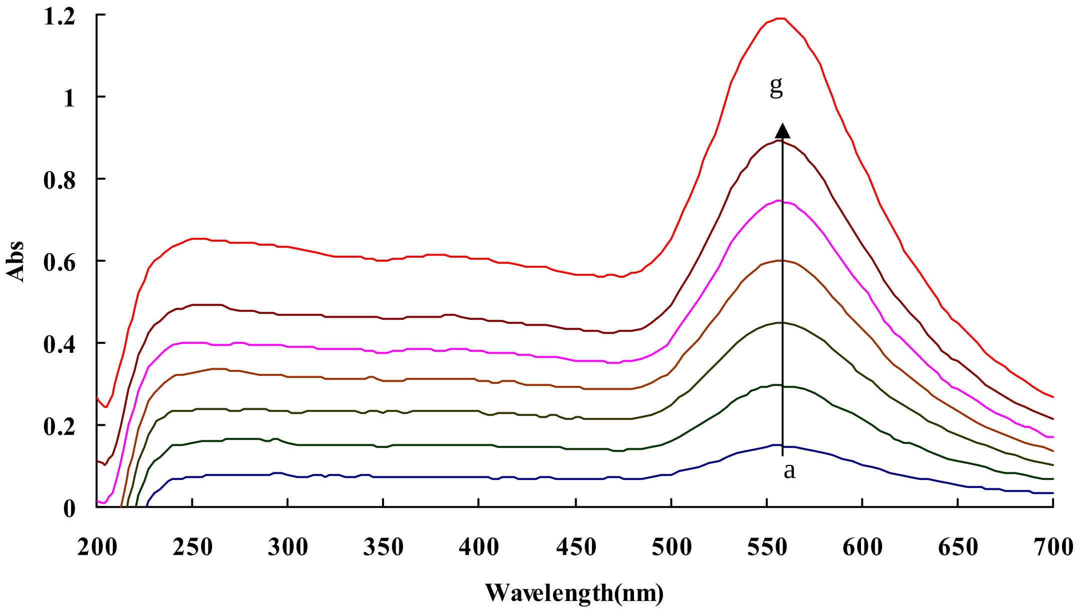


Fig. S1(a) Ultraviolet absorption spectra of AuNPs

a: 0.039 mmoL/L AuNPs; b: 0.078 mmoL/L AuNPs; c: 0.117 mmoL/L AuNPs; d:0.156mmoL/L AuNPs; e: 0.195mmoL/L AuNPs; f: 0.234 mmoL/L AuNPs; g: 0.293mmoL/L AuNPs.

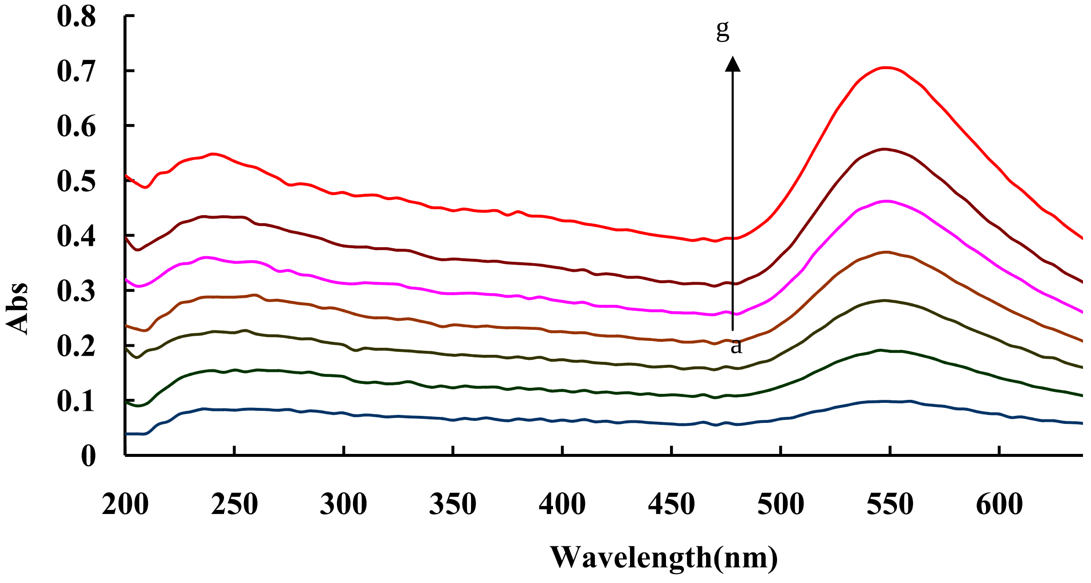


Fig. S1(b) Ultraviolet absorption spectra of Au/AuNPs

a: 0.039 mmoL/L Au/AuNPs; b: 0.078 mmoL/L Au/AuNPs ; c: 0.117 mmoL/L Au/AuNPs; d:0.156 mmoL/L Au/AuNPs; e: 0.195 mmoL/L Au/AuNPs; f: 0.234 mmoL/L Au/AuNPs; g: 0.293 mmoL/L Au/AuNPs.

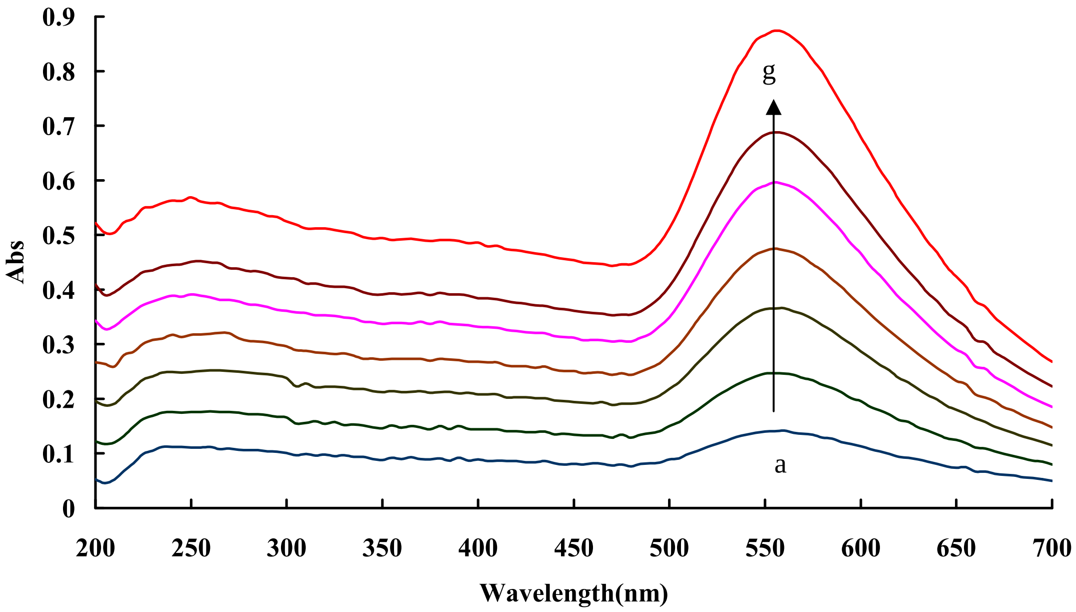


Fig. S1(c) Ultraviolet absorption spectra of Ag/AuNPs

a: 0.039 mmoL/L Ag/AuNPs; b: 0.078 mmoL/L Ag/AuNPs ; c: 0.117 mmoL/L Ag/AuNPs; d: 0.156 mmoL/L Ag/AuNPs; e: 0.195 mmoL/L Ag/AuNPs; f: 0.234 mmoL/L Ag/AuNPs; g: 0.293 mmoL/L Ag/AuNPs.

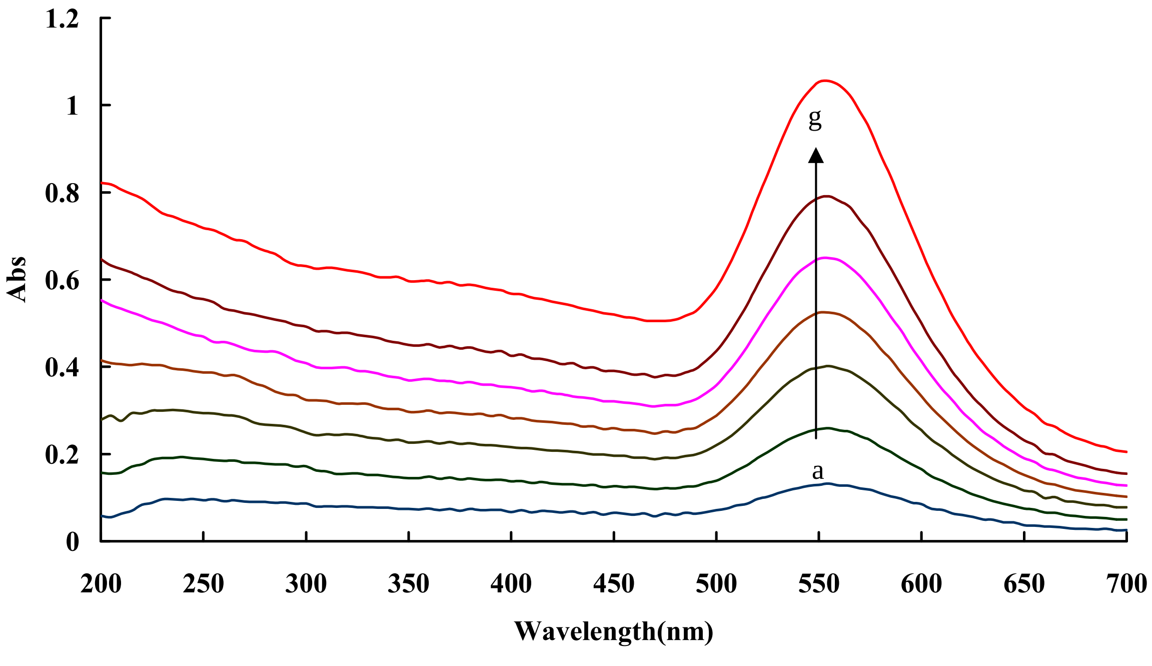


Fig. S1(d) Ultraviolet absorption spectra of GO/AuNPs

a: 0.039 mmoL/L GO/AuNPs;b: 0.078 mmoL/L GO/AuNPs ; c: 0.117 mmoL/L GO/AuNPs; d: 0.156 mmoL/L GO/AuNPs; e: 0.195 mmoL/L GO/AuNPs; f:0.234 mmoL/L GO/AuNPs; g: 0.293 mmoL/L GO/AuNPs.

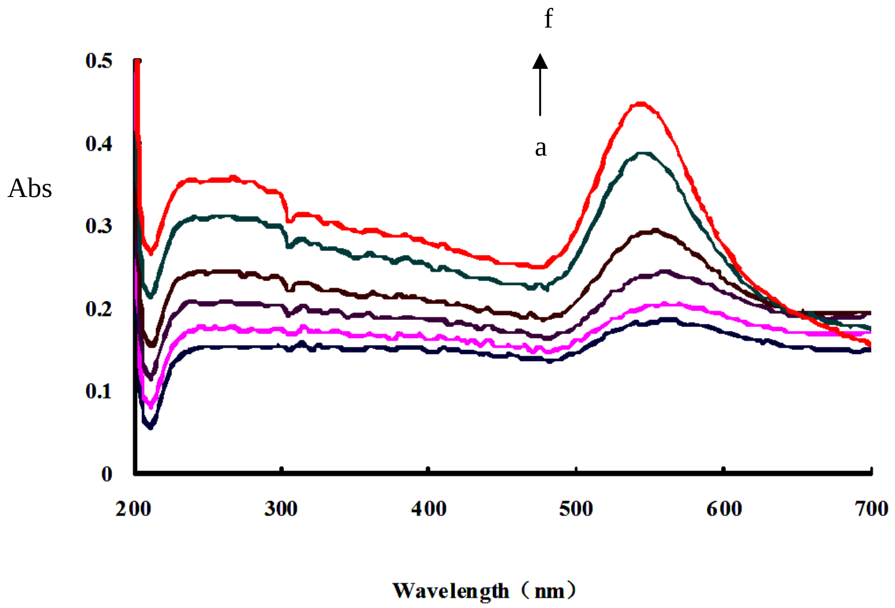


Fig. S2(a) Ultraviolet absorption spectra of AuNPs-HS-VB4r system

a: pH 4.0 20 mmoL/L NaAc-HAc +0.156 mmoL/L AuNPs +0.1 μmoL/L VB4r; b: a+5 ng/mLHS; c:a+25 ng/mLHS; d: a+50 g/mLHS; e: a+100 ng/mLHS;f: a+200 ng/mL HS.

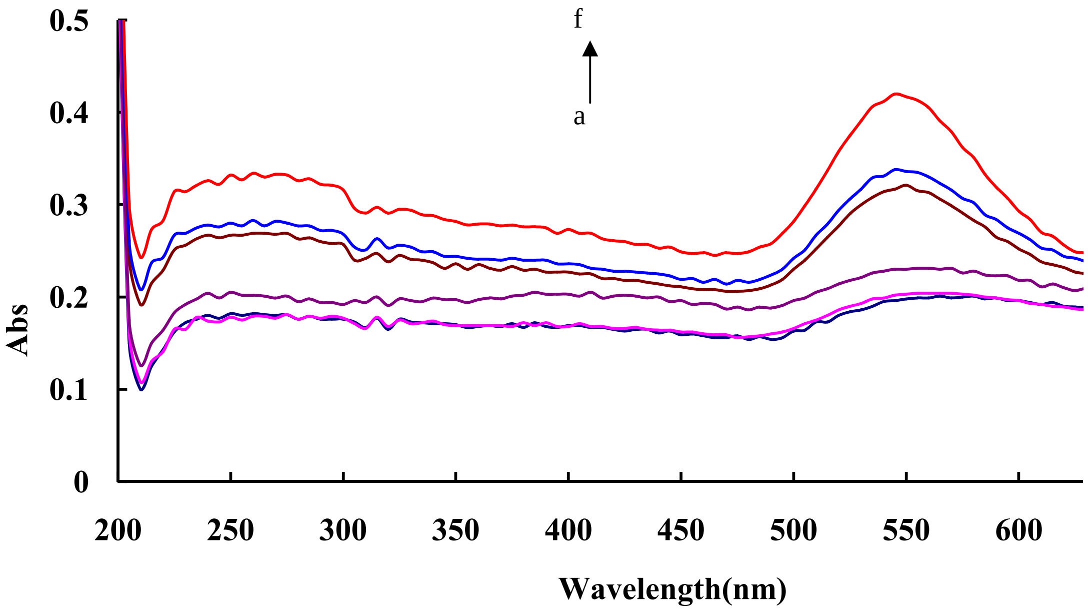


Fig. S2(b) Ultraviolet absorption spectra of Au/AuNPs-HS-VB4r system

a: pH 4.0 20 mmoL/L NaAc-HAc +0.195 mmoL/L Au/AuNPs +0.1 μmoL/L VB4r; b: a+1.25 ng/mL HS; c: a+25 ng/mL HS; d: a+50 ng/mL HS; e: a+100 ng/mL HS; f: a+150 ng/mL HS.

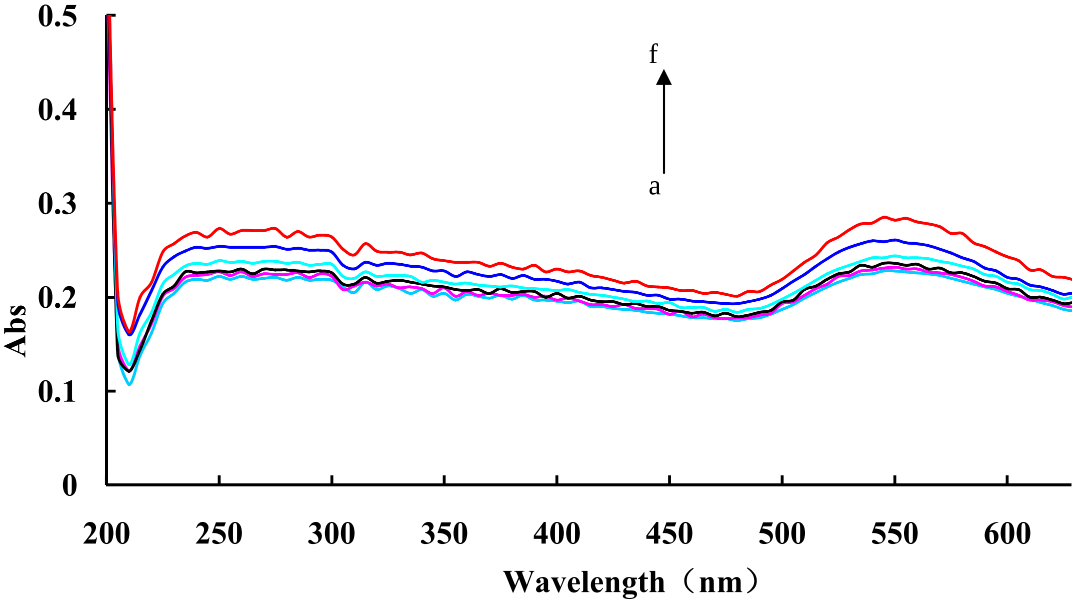


Fig. S2(c) Ultraviolet absorption spectra of Ag/AuNPs-HS-VB4r system
a: pH 4.0 20 mmoL/L NaAc-HAc +0.156 mmoL/L Ag/AuNPs +0.1 μmoL/L VB4r; b: a+5 ng/mL HS; c: a+25 ng/mL HS; d: a+50 ng/mL HS; e: a+100 ng/mL HS; f: a+200 ng/mL HS.

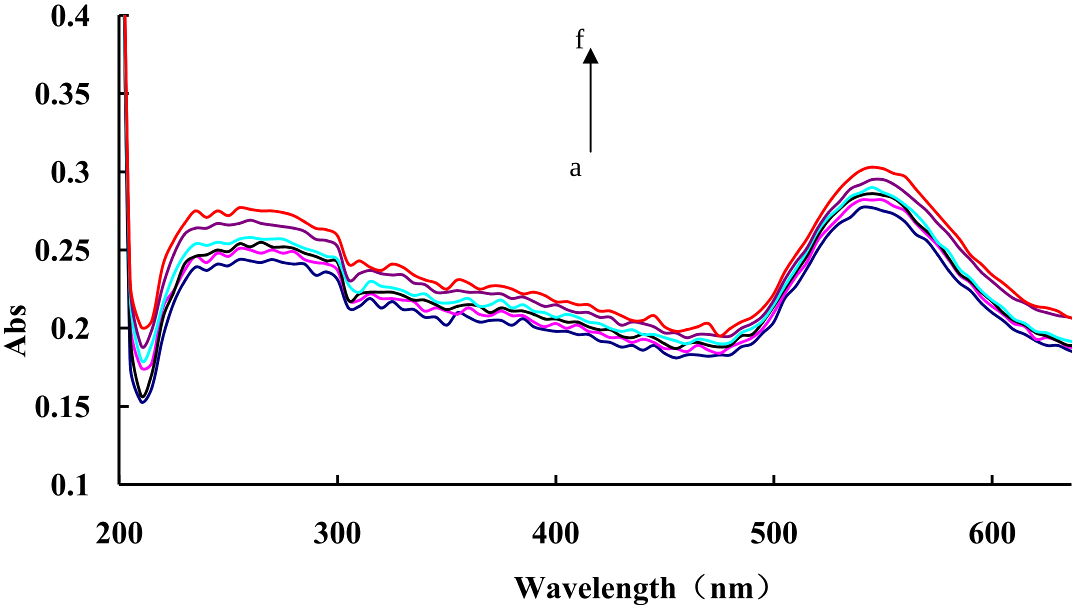


Fig. S2(d) Ultraviolet absorption spectra of GO/AuNPs-HS-VB4r system
a: pH 4.0 20 mmoL/L NaAc-HAc +0.156 mmoL/L GO/AuNPs +0.1 μmoL/L VB4r; b: a+2.5 ng/mL HS; c: a+25 ng/mL HS; d: a+50 ng/mL HS; e: a+100 ng/mL HS; f: a+150 ng/mL HS.

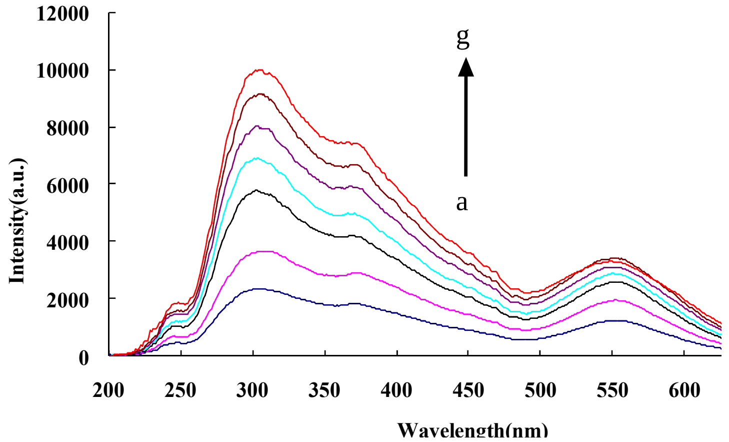


Fig. S3(a) RRS specta of AuNPs

a: 0.039 mmoL/L AuNPs; b: 0.078 mmoL/L AuNPs; c: 0.117 mmoL/L AuNPs; d: 6.8 mmoL/L AuNPs; e: 8.5 mmoL/L AuNPs; f: 10.2 mmoL/L AuNPs; g: 13.6 mmoL/L AuNPs.

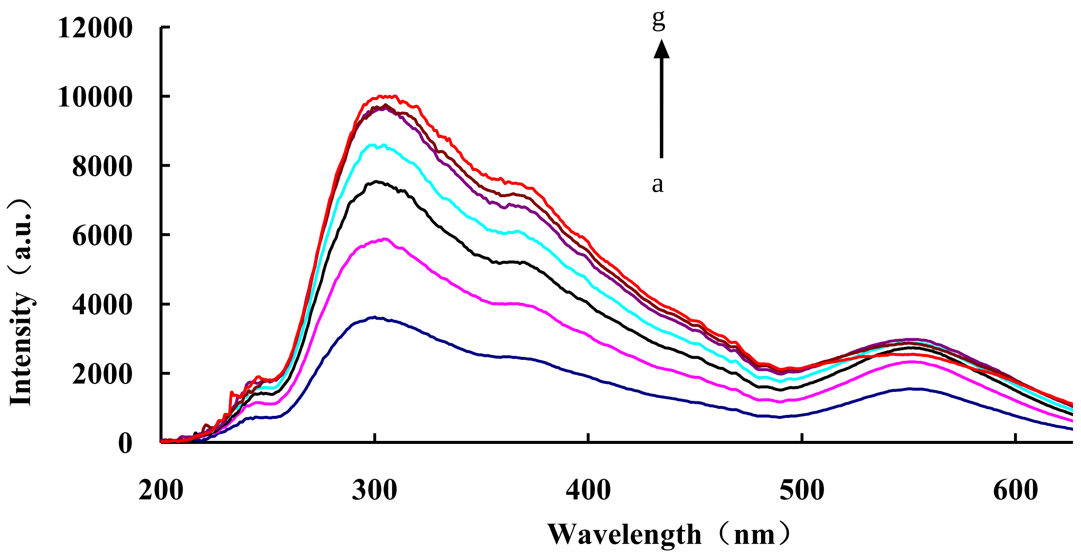


Fig. S3(b) RRS spectra of Au/AuNPs

a: 0.039 mmoL/L Au/AuNPs; b: 0.078 mmoL/L Au/AuNPs; c: 0.117 mmoL/L Au/AuNPs; d: 6.8 mmoL/L Au/AuNPs; e: 8.5 mmoL/L Au/AuNPs; f: 10.2 mmoL/L Au/AuNPs; g: 13.6 mmoL/L Au/AuNPs.

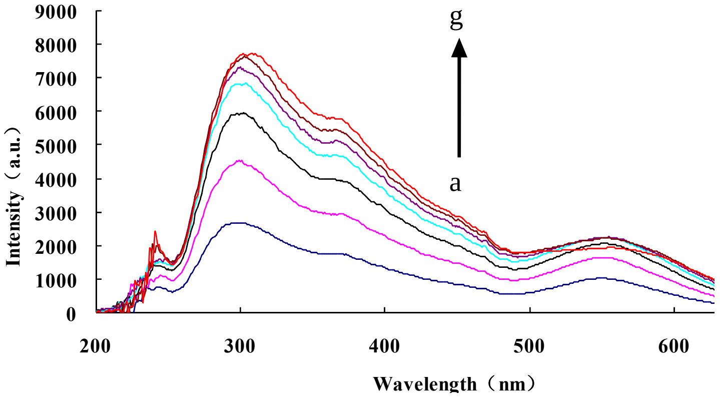


Fig. S3(c) RRS spectra of Ag/AuNPs

a: 0.039 mmoL/L Ag/AuNPs; b: 0.078 mmoL/L Ag/AuNPs; c:0.117 mmoL/L Ag/AuNPs; d: 0.156 mmoL/L Ag/AuNPs; e: 0.195 mmoL/L Ag/AuNPs; f: 0.234 mmoL/L Ag/AuNPs; g: 0.293 mmoL/L Ag/AuNPs.

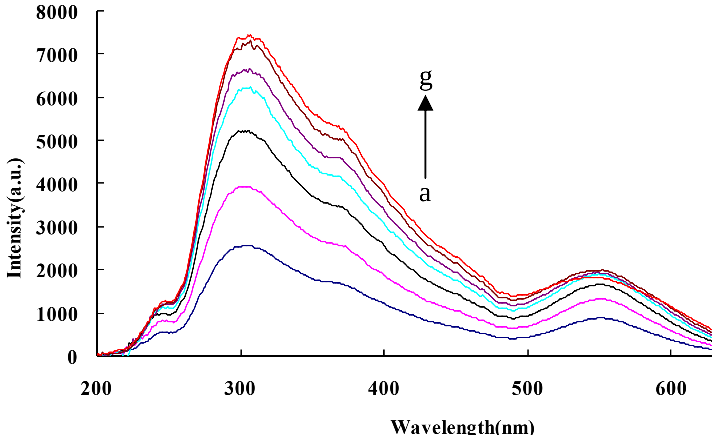


Fig. S3(d) RRS spectra of GO/AuNPs

a: 0.039 mmoL/L GO/AuNPs; b: 0.078 mmoL/L GO/AuNPs; c: 0.117 mmoL/L GO/AuNPs; d: 0.156 mmoL/L GO/AuNPs; e: 0.195 mmoL/L GO/AuNPs; f: 0.234 mmoL/L GO/AuNPs; g: 0.293 mmoL/L GO/AuNPs.

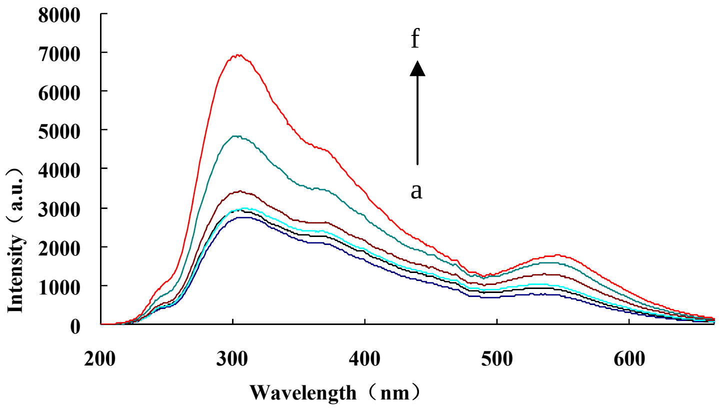


Fig. S4(a) RRS spectra of AuNPs-HS-VB4r system

a: pH 4.0 20 mmoL/L NaAc-HAc +0.156 mmoL/L AuNPs +0.1 μmoL/L VB4r; b: a+5 ng/mL HS; c: a+25 ng/mL HS; d: a+50 g/mL HS; e: a+100 ng/mL HS; f: a+200 ng/mL HS.

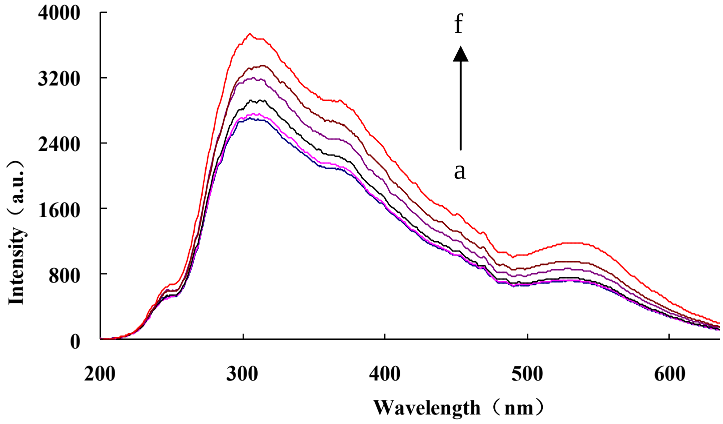


Fig. S4(b) RRS spectra of Ag/AuNPs-HS-VB4r system

a: pH 4.0 20 mmoL/L NaAc-HAc +0.156 mmoL/L Ag/AuNPs +0.1 μmoL/L VB4r; b: a+5 ng/mL HS; c: a+25 ng/mL HS; d: a+50 ng/mL HS; e: a+100 ng/mL HS; f: a+200 ng/mL HS.

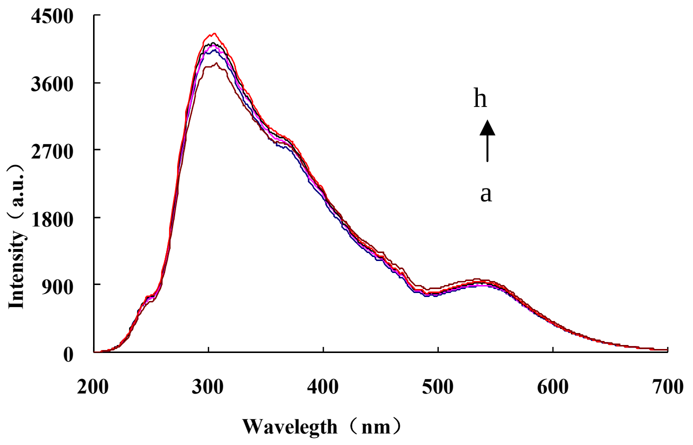

Fig. S4(c) RRS spectra of GO/AuNPs-HS-VB4r system

a: pH 4.0 20 mmoL/L NaAc-HAc +0.156mmoL/L GO/AuNPs +0.1μmoL/L VB4r; b: a+2.5 ng/mL HS; c: a+25 ng/mL HS; d: a+50 ng/mL HS; e: a+100 ng/mL HS; f: a+150 ng/mL HS.

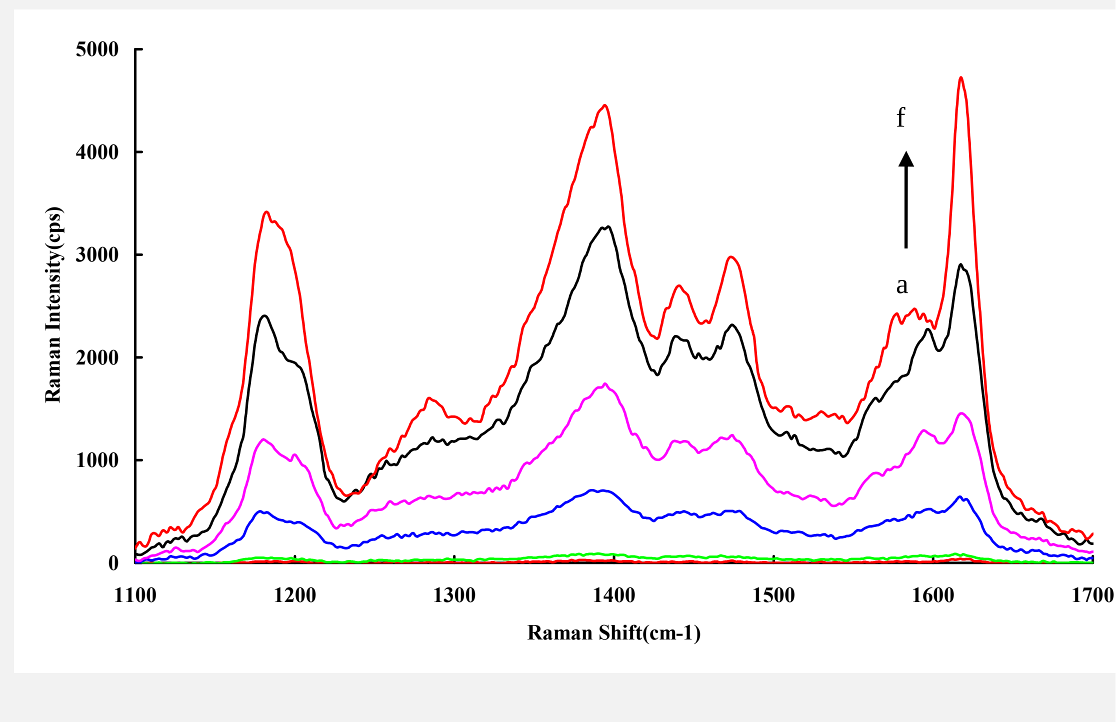


Fig. S5(a) SERS of the AuNPs

a：0.39 mmol/L AuNPs+25 mmoL/L NaCl; b: a +0.01μmoL/L VB4r; c: a+0.025 μmoL/L VB4r; d: a+0.05 μmoL/L VB4r; e: a+0.1 μmoL/L VB4r; f: a+0.2 μmoL/L VB4r.

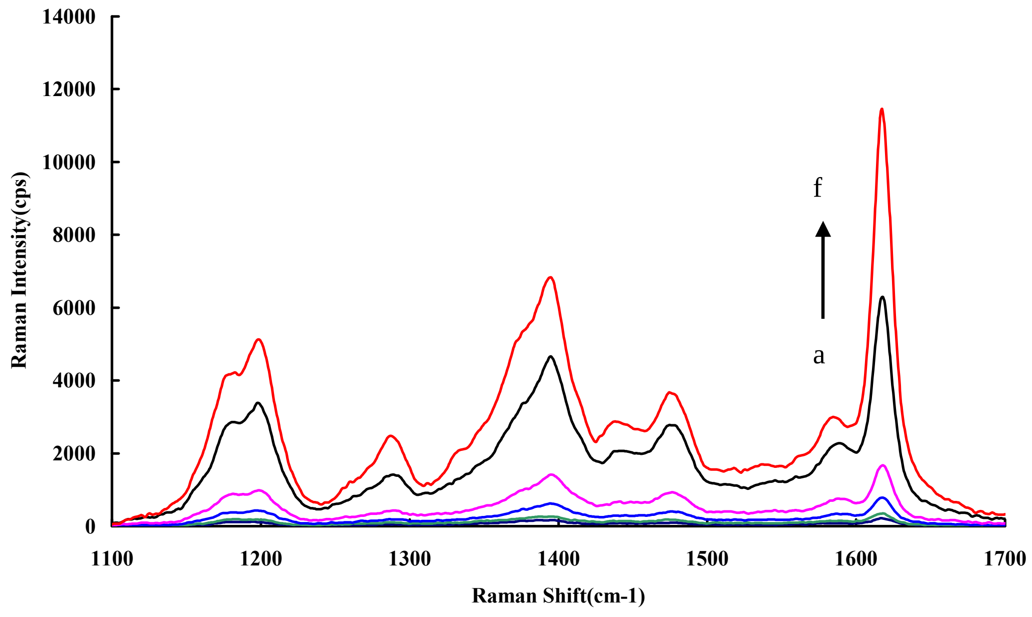


Fig. S5(b) SERS of Au/AuNPs

a: 0.39 mmol/L Au/AuNPs+25 mmoL/L NaCl; b: a +0.01 μmoL/L VB4r; c: a+0.025 μmoL/L VB4r; d: a+0.05 μmoL/L VB4r; e: a+0.1 μmoL/L VB4r; f: a+0.2 μmoL/L VB4r.

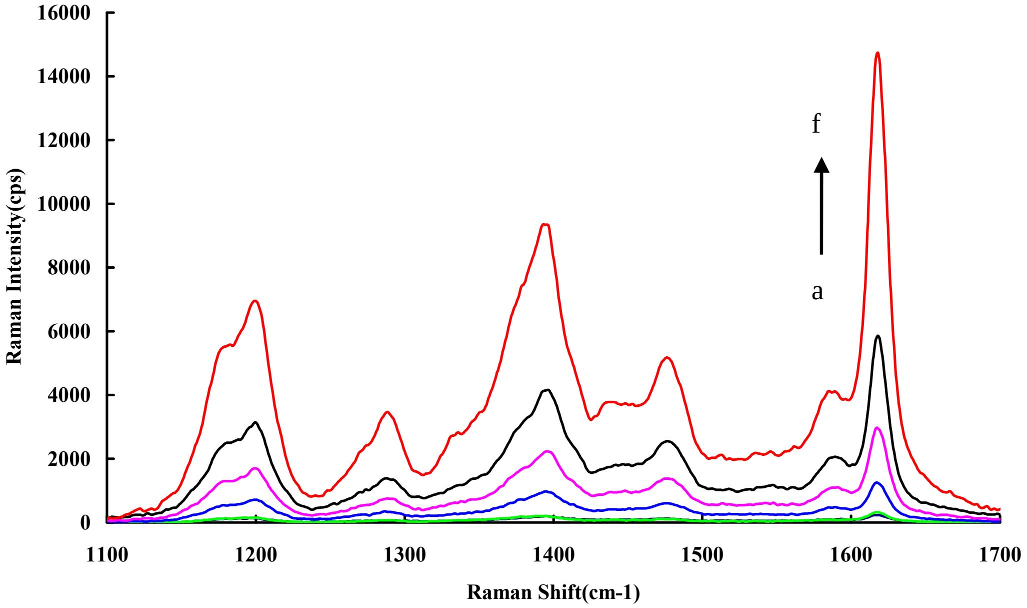


Fig. S5(c) SERS of Ag/AuNPs

a: 0.39 mmol/L Ag/AuNPs+25 mmoL/L NaCl; b: a +0.01 μmoL/L VB4r; c: a+0.025 μmoL/L VB4r; d: a+0.05 μmoL/L VB4r; e: a+0.1 μmoL/L VB4r; f: a+0.2 μmoL/L VB4r.

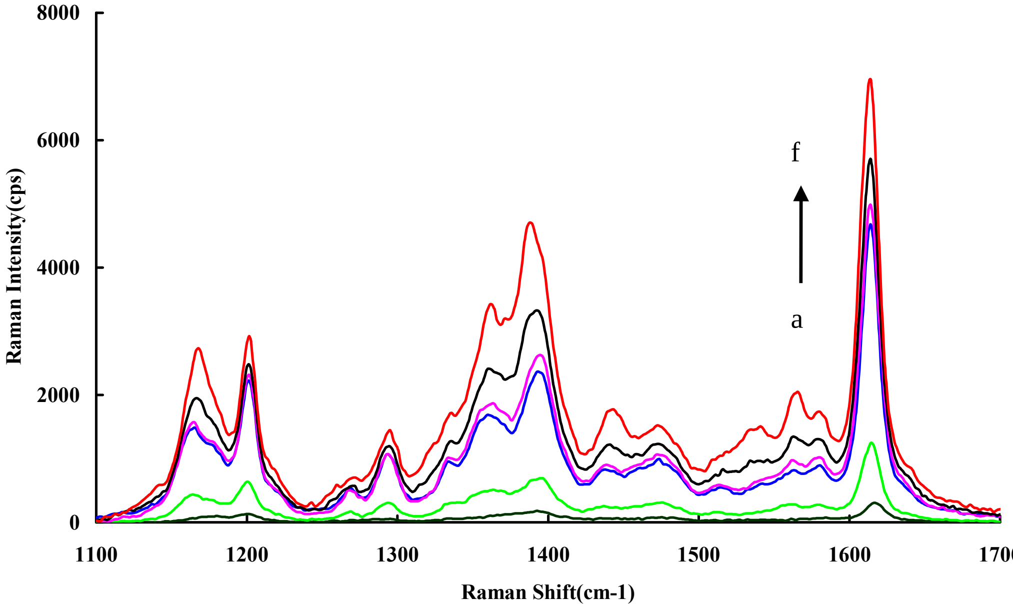


Fig. S5(d) SERS of GO/AuNPs

a: 0.39 mmol/L GO/AuNPs+25 mmoL/L NaCl; b: a +0.01 μmoL/L VB4r; c: a+0.025 μmoL/L VB4r; d: a+0.05 μmoL/L VB4r; e: a+0.1 μmoL/L VB4r; f: a+0.2 μmoL/L VB4r.

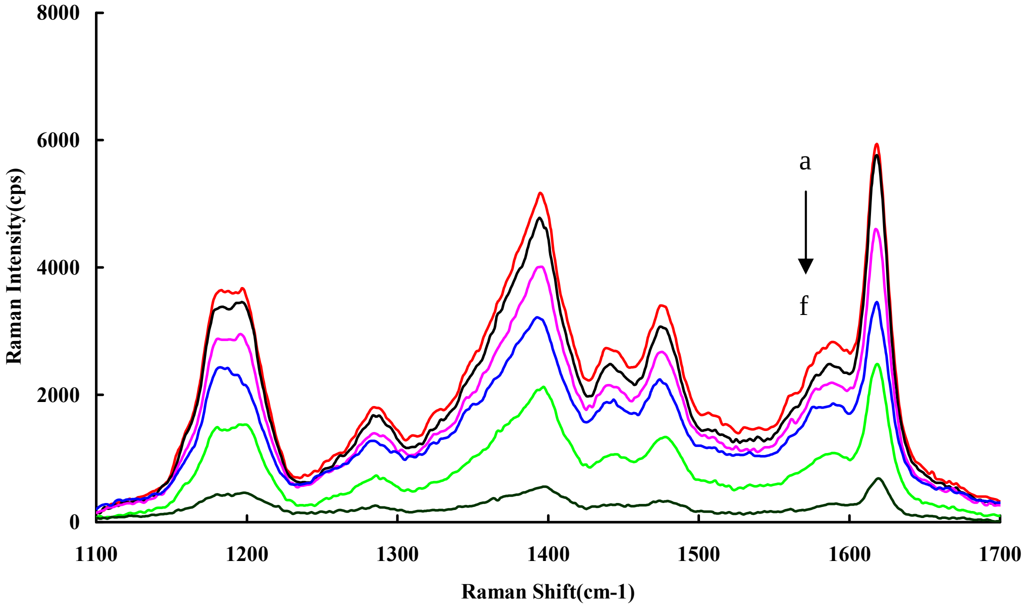


Fig. S6(a) SERS of AuNPs-HS-VB4r system

a: pH 4.0 20 mmoL/L NaAc-HAc+0.25 μmoL/L VB4r+0.156 mmoL /L AuNPs; b: a+5 ng/mLHS; c: a+25 ng/mLHS; d: a+50 g/mLHS; e: a+100 ng/mLHS; f: a+200 ng/mL HS.

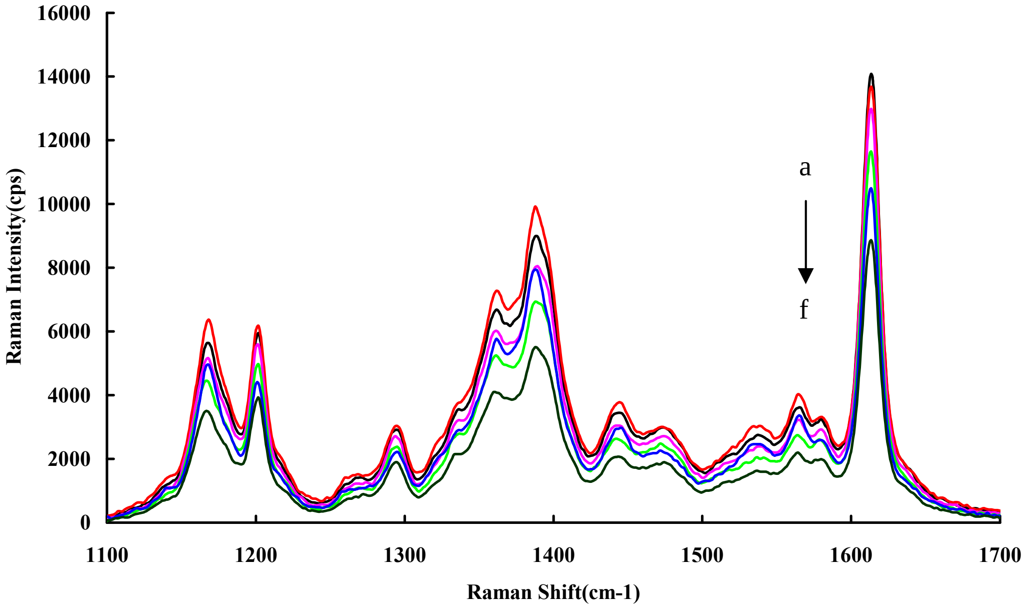


Fig. S6(b) SERS of Au/AuNPs-HS-VB4r system

a: pH 4.0 20 mmoL/L NaAc-HAc+0.1 μmoL/L VB4r+0.195 mmoL/L Au/AuNPs; b: a+1.25 ng/mL HS; c: a+12.5 ng/mL HS; d: a+25 ng/mL HS;e: a+50 ng/mL HS;f:

a+100 ng/mL HS.

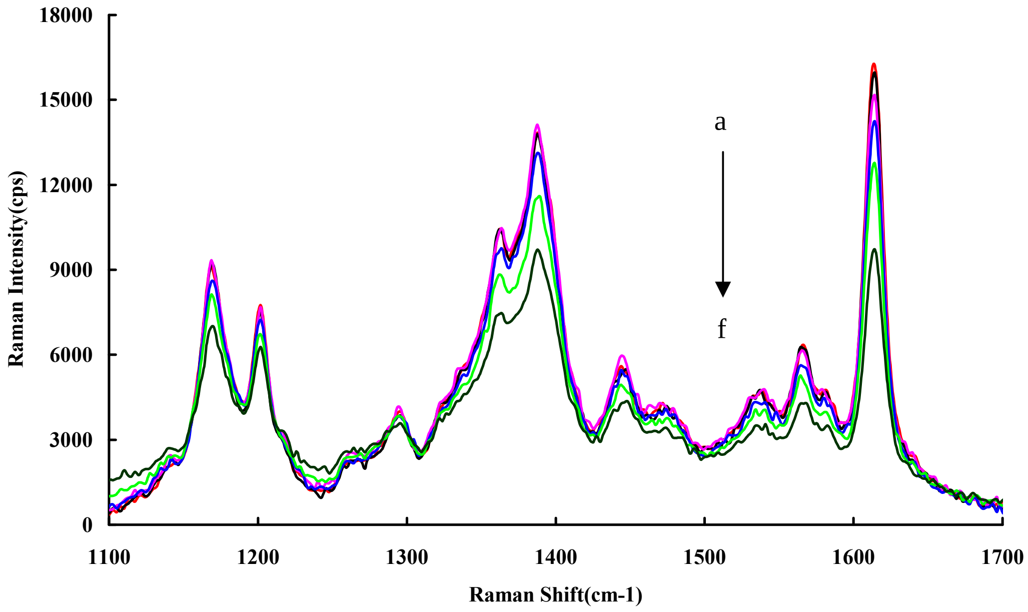


Fig. S6(c) SERS of Au/AuNPs-HS-VB4r-$AlCl_3$ System

a: pH 4.0 20 mmoL/L NaAc-HAc+0.1 μmoL/L VB4r+0.195 mmoL/L Au/AuNPs+1.5 μmoL/L $AlCl_3$; b: a+1.25 ng/mL HS; c: a+25 ng/mL HS; d: a+50 ng/mL HS; e: a+100 ng/mL HS; f: a+150 ng/mL HS.

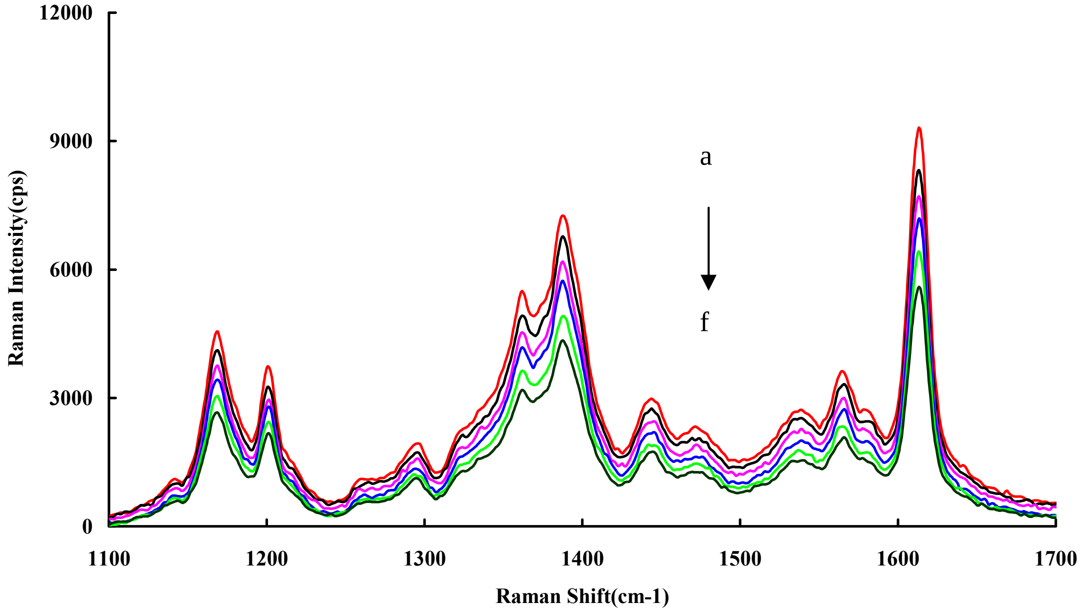


Fig.S6(d) SERS of Ag/AuNPs-HS-VB4r system

a: pH 4.0 20 mmoL/L NaAc-HAc+0.1 μmoL/L VB4r+0.156 mmoL/L Ag/AuNPs; b: a+5 ng/mL HS; c: a+25 ng/mL HS; d: a+50 ng/mL HS; e: a+100 ng/mL HS; f: a+200 ng/mL

HS.

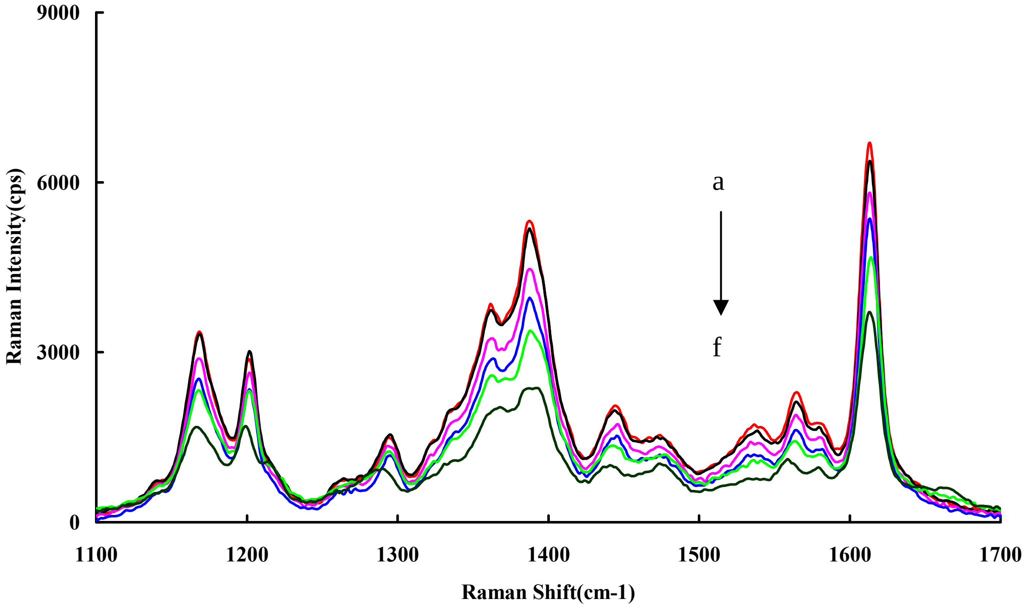


Fig.S6(e) SERS of GO/AuNPs-HS-VB4r system

a: pH 4.0 20 mmoL/L NaAc-HAc+0.1 μmoL/L VB4r+0.156 mmoL/L GO/AuNPs; b: a+2.5 ng/mL HS; c: a+25 ng/mL HS; d: a+50 ng/mL HS; e: a+100 ng/mL HS; f: a+150 ng/mL HS.

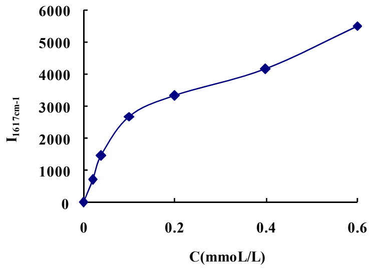


Fig.S7 Effect of *L*-CyS concentration on the reaction of *L*-CyS-$HAuCl_4$

*L*-CyS+0.39 mmoL/L $HAuCl_4$

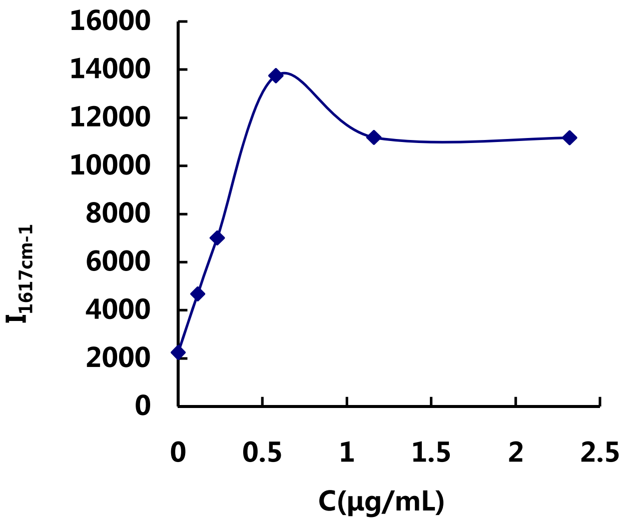


Fig.S8(a) Effect of AuNPc concentration on the reaction of *L*-CyS-$HAuCl_4$

AuNPc+ 0.1 mmoL/L *L*-CyS+0.39 mmoL/L $HAuCl_4$

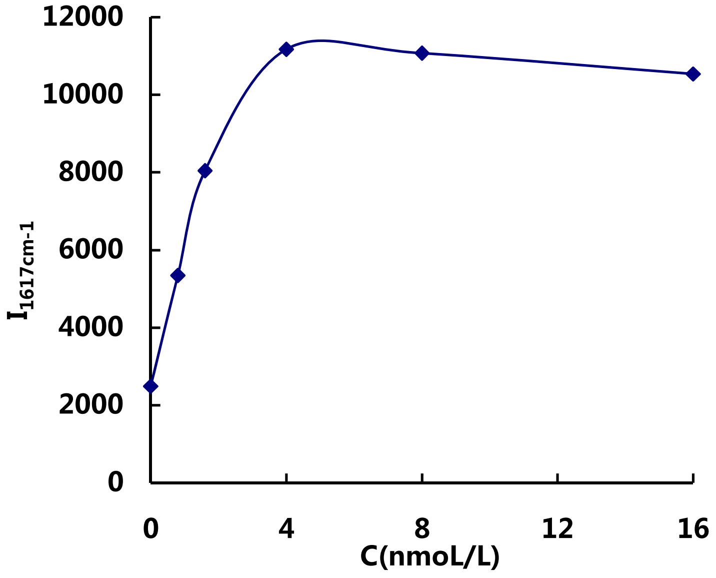


Fig. S8(b) Effect of AgNPs concentration on the reaction of *L*-CyS-$HAuCl_4$

AgNPs+ 0.1 mmoL/L *L*-CyS+0.39 mmoL/L $HAuCl_4$

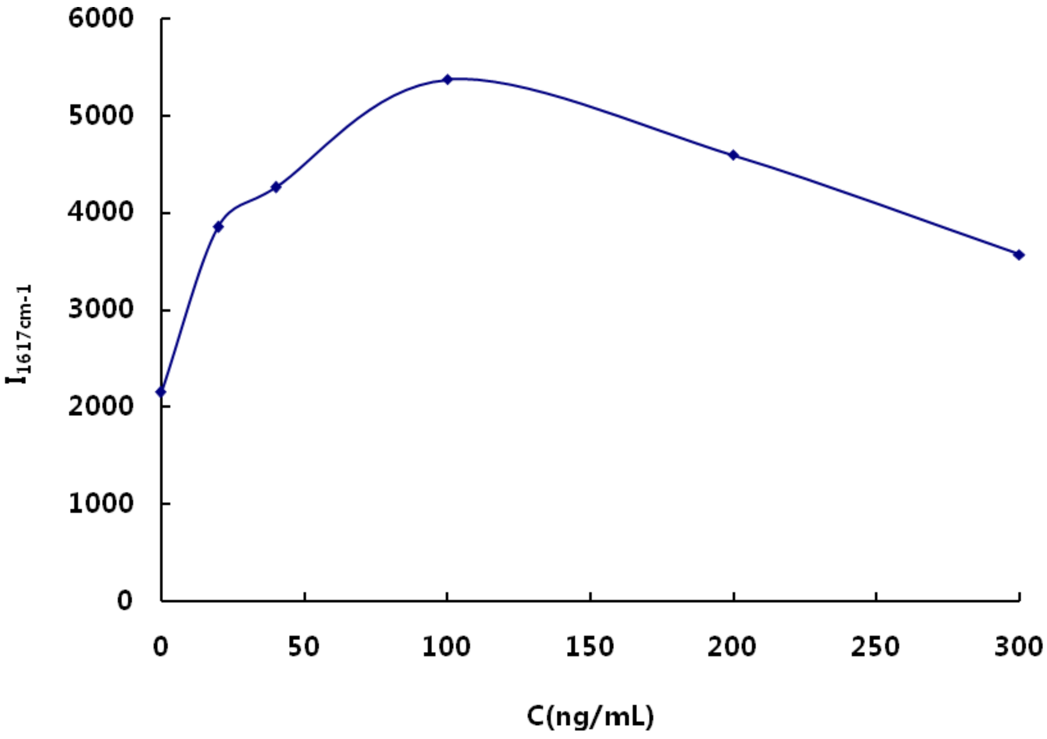


Fig. S8(c) Effect of GO concentration on the reaction of *L*-CyS-$HAuCl_4$

GO+ 0.1 mmoL/L *L*-CyS+0.39 mmoL/L $HAuCl_4$

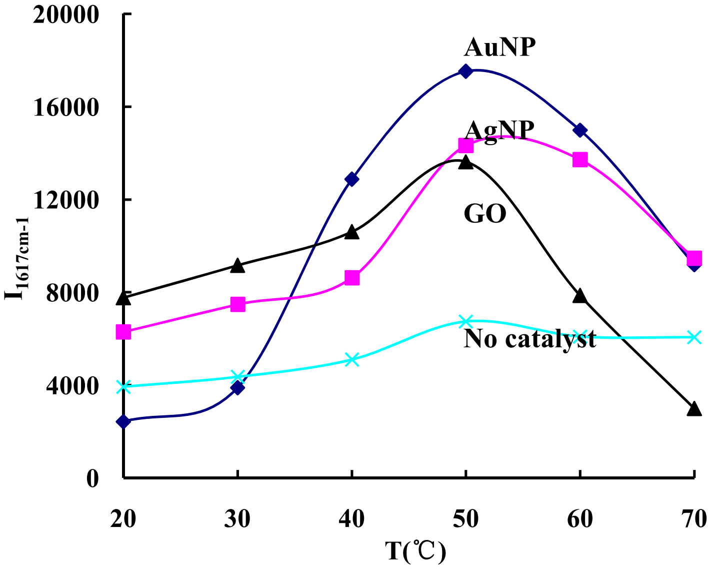


Fig. S9 Effect of temperature on the reaction of *L*-CyS-$HAuCl_4$

nanoatalyst+ 0.1 mmoL/L *L*-CyS+0.39 mmoL/L $HAuCl_4$

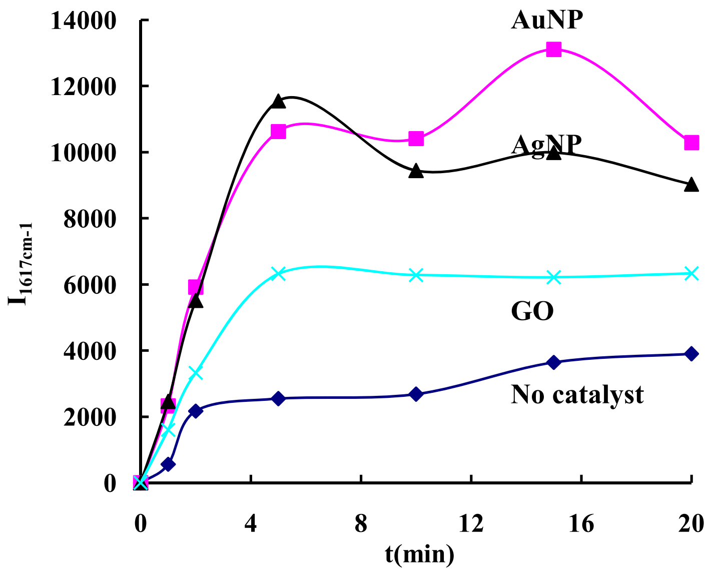


Fig.S10 Effect of time on the reaction of *L*-CyS-$HAuCl_4$

nanoatalyst+ 0.1 mmoL/L *L*-CyS+0.39 mmoL/L $HAuCl_4$

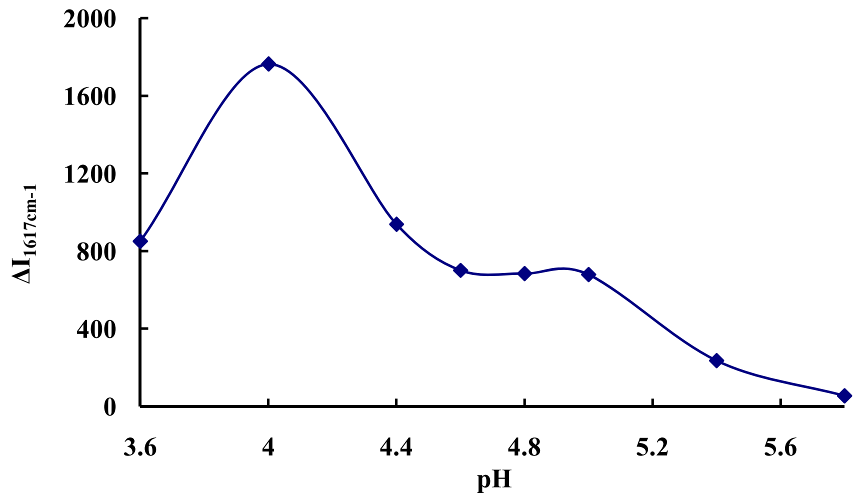


Fig. S11 Effect of pH on $\Delta I_{1617cm\text{-}1}$ of the analytical system

5 mmoL/L NaAc-HAc+0.195 mmoL/L Au/AuNPs+0.25 μmoL/L VB4r+100 ng/mL HS

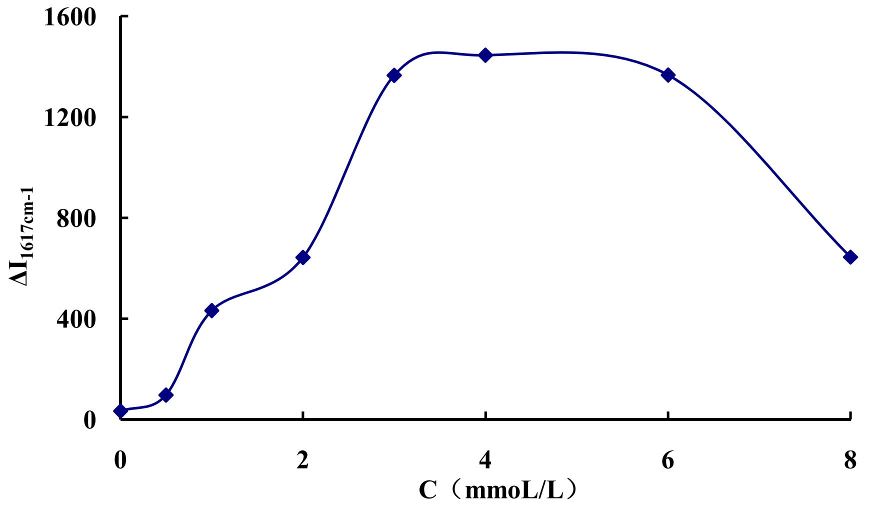


Fig. S12 Effect of buffer solution concentration on $\Delta I_{1617cm\text{-}1}$ of the analytical system

pH4.0 NaAc-HAc+0.195 mmoL/L Au/AuNPs+0.25 μmoL/L VB4r+100 ng/mL HS

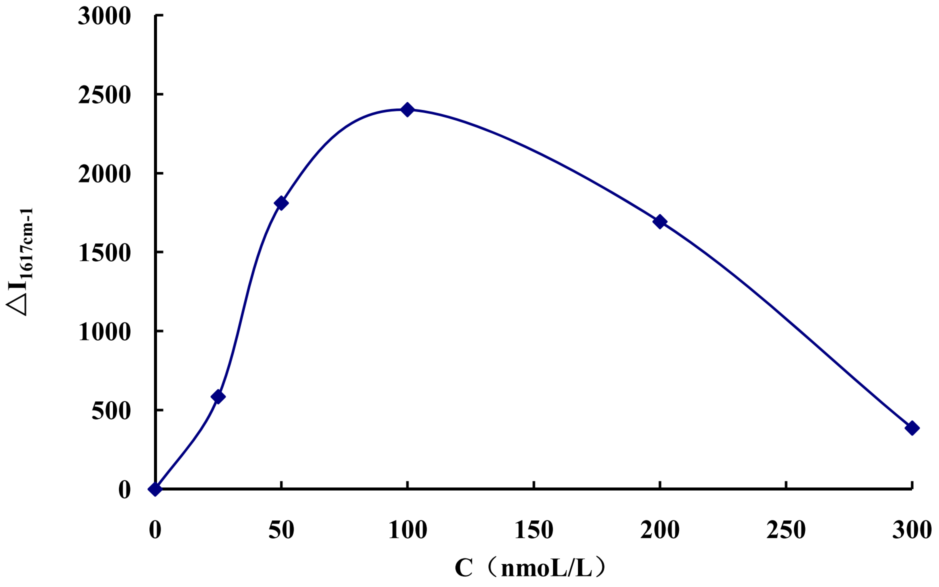


Fig. S13 Effect of VB4r concentration on $\Delta I_{1617cm\text{-}1}$ of the analytical system

pH 4.0 NaAc-HAc+0.195 mmoL/L Au/AuNPs+100 ng/mL HS

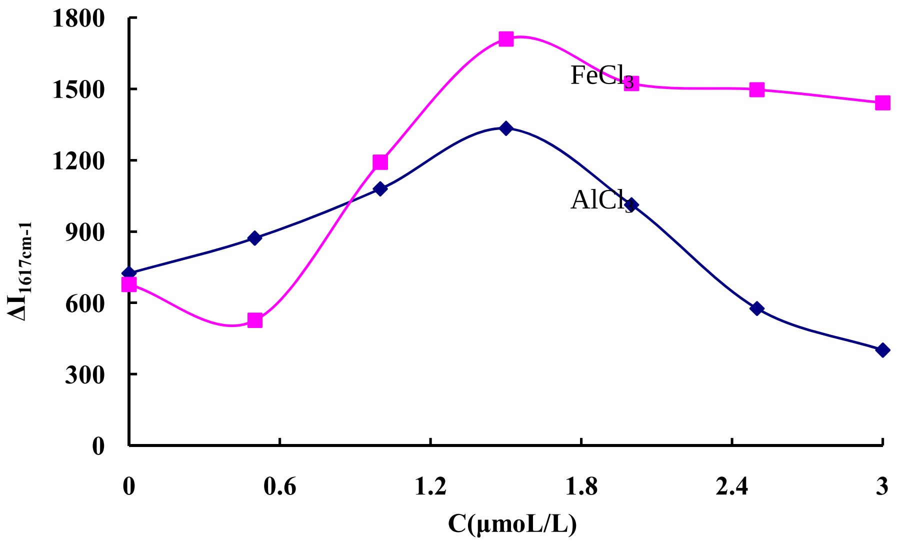


Fig. S14 Effect of sensitizer concentration on $\Delta I_{1617cm-1}$ of the analytical system
0.195 mmoL/L Au/AuNPs+ 0.1 μmoL/L VB4r+100 ng/mL HS+ sensitizer

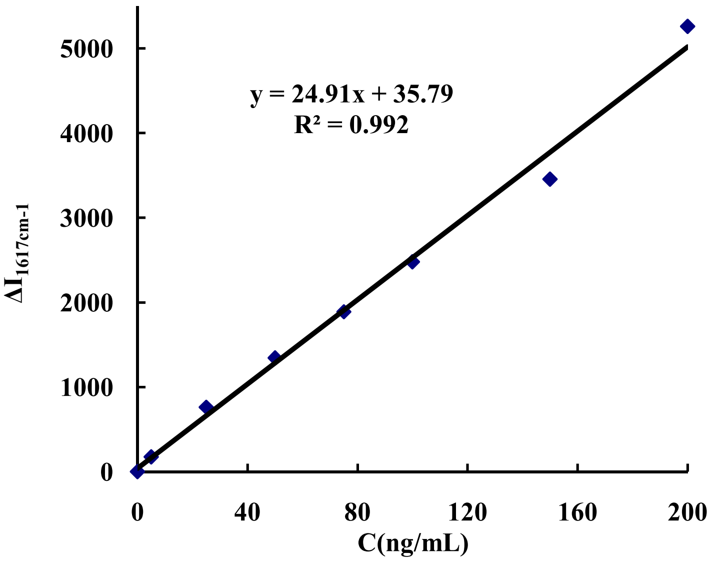


Fig. S15(a) Standard curve of AuNPs-HS-VB4r system
pH4.0 NaAc-HAc+0.156 mmoL/L AuNPs +0.1 μmoL/L VB4r

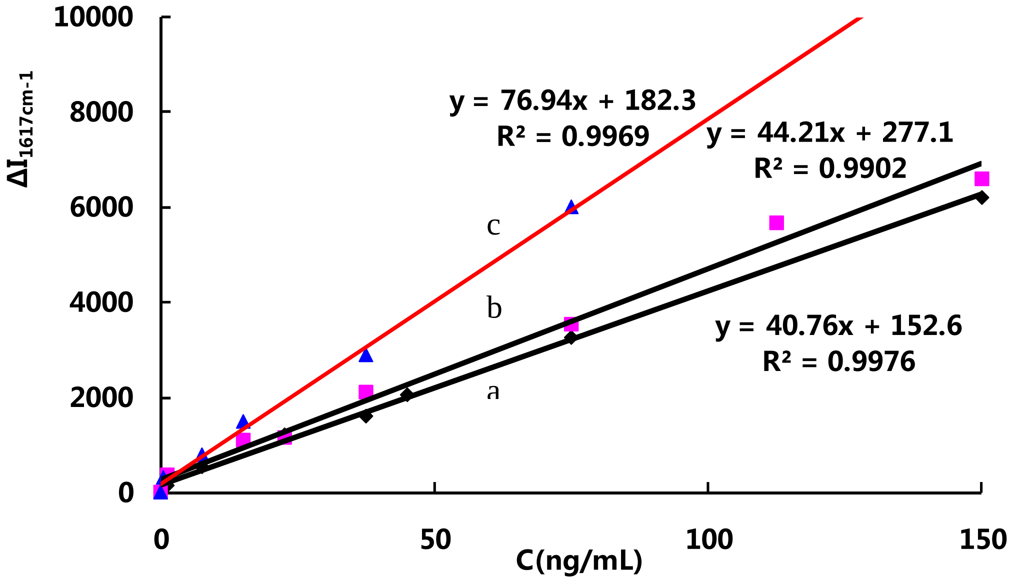


Fig. S15(b) Standard curve of Au/AuNPs-HS-VB4r system

(a) pH4.0 NaAc-HAc+0.195 mmoL/L Au/AuNPs +0.1 μmoL/L VB4r; (b) a+$AlCl_3$ ; (c) a+$FeCl_3$.

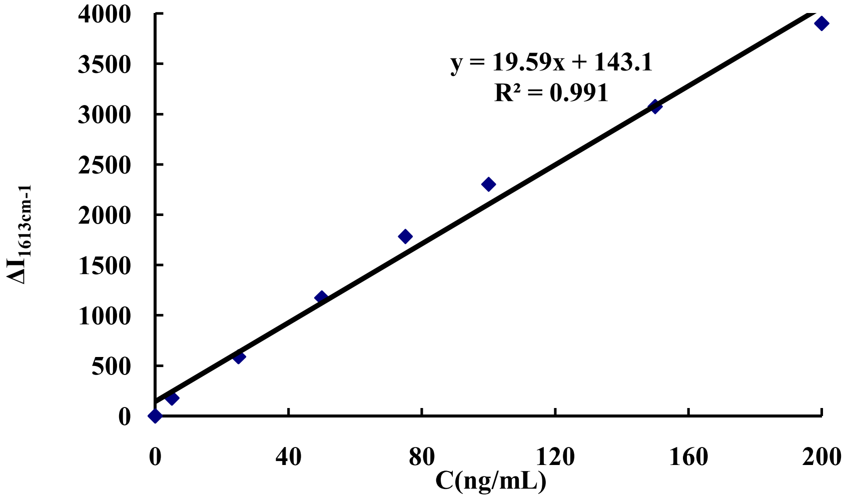


Fig. S15(c) Standard curve of Ag/AuNPs-HS-VB4r system

pH4.0 NaAc-HAc+0.156 mmoL/L Ag/AuNPs +0.1 μmoL/L VB4r

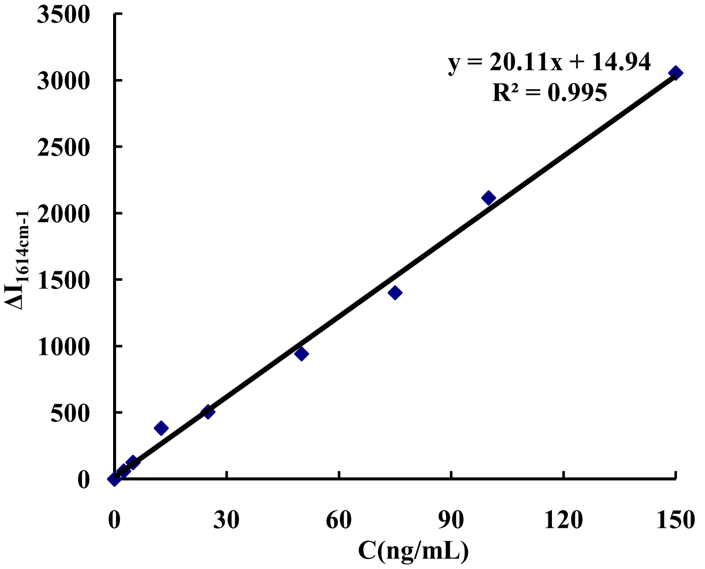


Fig. S15(d) Standard curve of GO/AuNPs-HS-VB4r system

pH4.0 NaAc-HAc+0.156 mmoL/L GO/AuNPs +0.1 μmoL/L VB4r

**Tab. S1 Stability of SERS signal ($I_{1617cm-1}$) of Synthesis of Nanosol with Different *L*-CyS Concentration**

| Concentration（mmoL/L） | 0.02 | 0.04 | 0.1 | 0.2 | 0.4 | 0.6 |
|---|---|---|---|---|---|---|
| Day 1 | 737.4 | 1375 | 2214 | 3126.8 | 3783 | 5389.2 |
| Day 2 | 633.8 | 1450.8 | 2591.8 | 3369.4 | 3664.4 | 5197 |
| Day 3 | 780.8 | 1470.8 | 2452.6 | 3298.6 | 4084.6 | 5201.2 |
| Day 4 | 718.4 | 1665.4 | 2642.4 | 3484.6 | 4086.4 | 6502 |
| Day 5 | 667.6 | 1608 | 2693.8 | 3436.6 | 5197 | 5292 |
| Average value | 707.6 | 1474.12 | 2698.92 | 3343.2 | 4163.08 | 5516.28 |
| RSD（%） | 8.18 | 8.09 | 7.14 | 4.18 | 14.58 | 10.09 |

**Tab. S2 Stability of AuNPs***

| Methods | Time | Day 1 | Day 2 | Day 3 | Day 5 | Day 7 | Average Value | RSD（%） |
|---|---|---|---|---|---|---|---|---|
| Abs | $A_{575}$ | 0.607 | 0.644 | 0.611 | 0.65 | 0.665 | 0.633 | 3.71 |
| RRS | $I_{550}$ | 3555 | 3628 | 2994 | 2987 | 3573 | 3347.4 | 9.77 |
| SERS | $I_{1617cm-1}$ | 5583.7 | 5368.3 | 4976 | 6126.0 | 6279.0 | 5666.6 | 8.49 |

* The concentration of Au/AuNPs was 0.39 mmol/L

**Tab. S3 Stability of Au/AuNPs***

| Methods | Time | Day 1 | Day 2 | Day 3 | Day 5 | Day 7 | Average Value | RSD （%） |
|---|---|---|---|---|---|---|---|---|
| Abs | $A_{555}$ | 0.352 | 0.331 | 0.331 | 0.39 | 0.302 | 0.3412 | 9.55 |
| RRS | $I_{550}$ | 2094 | 2072 | 1737 | 2005 | 1872 | 1956 | 7.67 |
| SERS | $I_{1617cm-1}$ | 9836 | 10352 | 10070 | 10491 | 10626 | 10275 | 3.12 |

* The concentration of AuNPs was 0.39 mmol/L

**Tab. S4 Stability of Ag/AuNPs***

| Methods | Time | Day 1 | Day 2 | Day 3 | Day 5 | Day 7 | Average Value | RSD （%） |
|---|---|---|---|---|---|---|---|---|
| Abs | $A_{575}$ | 0.315 | 0.304 | 0.292 | 0.331 | 0.27 | 0.3024 | 7.64 |
| RRS | $I_{550}$ | 2460 | 2367 | 1962 | 2333 | 2196 | 2263.6 | 8.57 |
| SERS | $I_{1617cm-1}$ | 13937 | 14358 | 14166 | 15344 | 16138 | 14788.6 | 6.26 |

* The concentration of Ag/AuNPs was 0.39 mmol/L

**Tab. S5 Stability of GO/AuNPs***

| Methods | Time | Day 1 | Day 2 | Day 3 | Day 5 | Day 7 | Average Value | RSD （%） |
|---|---|---|---|---|---|---|---|---|
| Abs | $A_{555}$ | 1.316 | 1.298 | 1.348 | 1.371 | 1.294 | 1.3254 | 2.51 |
| RRS | $I_{550nm}$ | 1831 | 2026 | 1709 | 1876 | 1639 | 1816.2 | 8.29 |
| SERS | $I_{1617cm-1}$ | 6442.7 | 5328.3 | 6501.5 | 6567.8 | 6615.0 | 6291.1 | 7.71 |

* The concentration of GO/AuNPs was 0.39 mmol/L

**Tab S6 Compare of different preparation methods of nanoparticles**

| Method | Principle | Comment | Reference |
|---|---|---|---|
| Trisodium Citrate | Using sodium citrate to reduce chlorine acid | Stable, uniform, and low SERS activity | 36 |
| Sodium Borohydride | Using sodium borohydride to reduce chlorine acid with thiol succinic acid (N/A) as the protective agent | Stable, uniform, and low SERS activity, not environmentally friendly | 37 |
| Ascorbic Acid | Using ascorbic acid to reduce chlorine acid with thiol sodium citrate as the protective agent | Stable, uniform, and good SERS activity, Lower purity | 38 |
| Microwave | Using sodium citrate to reduce chlorine acid in the condition of microwave | Stable, uniform, and low SERS activity, not environmentally friendly | 39 |
| Photochemical | Using photochemical to reduce chlorine acid | Environmentally friendly, and slow speed, low efficiency | 40 |
| Microbial | Using saccharomycetes to reduce chlorine acid | Environmentally friendly, mild reaction and unstable and poor reproducibility | 41 |
| AuNP catalyst | Using *L*-CyS to reduce chlorine acid in catalyst of nanogold | Environmentally friendly, preparation of rapid, high efficiency, stable, uniform | The method |

**Table S7 Effect of interfering ions on Au/AuNPs-HS-VB4r system**

| Coexisting Substance | Magnification | Relative Error (%) | Coexisting Substance | Magnification | Relative Error (%) |
|---|---|---|---|---|---|
| $Cr^{6+}$ | 100 | -0.50 | $SO_3^{2-}$ | 1 | -2.42 |
| $Pb^{2+}$ | 100 | -6.41 | $NO_2^-$ | 100 | -5.16 |
| $K^+$ | 100 | -2.44 | $S_2O_3^{2-}$ | 10 | -7.59 |
| $Zn^{2+}$ | 100 | 1.54 | Oxalic Acid | 10 | -5.28 |
| $Mn^{2+}$ | 100 | -5.63 | Glycine | 100 | -2.62 |
| $Ca^{2+}$ | 100 | 2.84 | Glucose | 100 | 2.15 |
| $Co^{2+}$ | 100 | 0.50 | *L*-Valine | 100 | -10.09 |
| $Mg^{2+}$ | 100 | 1.54 | *L*-Threonine | 100 | 2.10 |
| $Ba^{2+}$ | 100 | 1.38 | Lauric acid | 100 | 3.12 |
| $N^{i2+}$ | 10 | 2.37 | Etearic acid | 100 | 2.25 |
| $Bi^{3+}$ | 10 | -2.54 | Leithin | 100 | -1.32 |
| $Fe^{3+}$ | 10 | -3.02 | CTAB | 100 | 9.86 |

**Tab. S8 Analysis results of HS samples**

| Sample | Added （ng/mL） | Found (ng/mL ) | Average (ng/mL ) | Titer (IU/mL) | RSD (%) | Recovery (%) |
|---|---|---|---|---|---|---|
| 1# | No | 34.55, 35.56, 32.63, 37.21，34.53 | 34.89 | 6280 | 4.79 | No |
| | 10 | 43.73, 45.22, 43.18, 46.09，45.11 | 44.67 | 8039 | 2.65 | 99.9 |
| 2# | No | 32.33, 33.32, 35.57, 33.12，34.22 | 33.72 | 6078 | 3.61 | No |
| | 20 | 53.18, 55.87, 56.57, 52.08，53.84， | 54.32 | 9779 | 3.50 | 99.3 |
| 3# | No | 36. 54, 38.79, 32.03, 34.96，34.18 | 33.9 | 6102 | 7.63 | No |
| | 30 | 64.56, 69.32, 63.25, 62.88，66.89 | 65.38 | 11840 | 4.13 | 101.0 |
